# NADH:ubiquinone oxidoreductase core subunit S8 expression and functional significance in non-small cell lung cancer

**DOI:** 10.1038/s41419-025-07638-5

**Published:** 2025-04-21

**Authors:** Weihua Xu, Hongpeng Fang, Xianbao Cao, Min-zhao Xu, Yubo Yan, Mingjing Shen, Yi Yang, Kanqiu Jiang

**Affiliations:** 1 Department of Thoracic and Cardiac Surgery, The Second Affiliated Hospital of Soochow University, Suzhou, China; 2 Department of Thoracic Surgery, Harbin Medical University Cancer Hospital, Harbin, China; 3 Department of Nuclear Medicine, the Affiliated Suzhou Science & Technology Town Hospital of Nanjing Medical University, Suzhou, China

**Keywords:** Non-small-cell lung cancer, Tumour biomarkers

## Abstract

Hyperfunctional mitochondria provide a growth advantage by supporting the energy-intensive processes essential for non-small cell lung cancer (NSCLC). NADH:ubiquinone oxidoreductase core subunit S8 (NDUFS8) is a key subunit of mitochondrial complex I involved in oxidative phosphorylation (OXPHOS) and cellular energy production. Bioinformatics and local tissue examinations show that NDUFS8 expression is elevated in NSCLC compared to normal lung tissue. Both immortalized and primary human NSCLC cells exhibit higher NDUFS8 levels. Single-cell RNA sequencing confirmed NDUFS8 upregulation in cancerous cells of NSCLC tumor. Silencing NDUFS8 via shRNA or Cas9/sgRNA-mediated knockout (KO) disrupted mitochondrial functions, leading to decreased complex I activity, ATP depletion, mitochondrial depolarization, increased reactive oxygen species (ROS) production, and heightened lipid peroxidation. Furthermore, NDUFS8 silencing/KO triggered apoptosis and significantly reduced Akt-mTOR activation, cell viability, proliferation, and motility in various NSCLC cells. In contrast, ectopic overexpression of NDUFS8 boosted mitochondrial complex I activity and ATP levels, promoting Akt-mTOR activation, and enhancing NSCLC cell proliferation and motility. NDUFS8 also contributes to radioresistance in NSCLC; silencing or KO enhanced ionizing radiation (IR)-induced cytotoxicity, while overexpression mitigated it. Intratumoral injection of NDUFS8 shRNA-expressing adeno-associated virus significantly inhibited growth of primary NSCLC xenografts in nude mice, with observed NDUFS8 silencing, ATP reduction, oxidative damage, proliferation inhibition, Akt-mTOR inactivation and apoptosis in treated tissues. These findings highlight the pivotal pro-tumorigenic role of NDUFS8 in NSCLC.

## Introduction

Non-small cell lung cancer (NSCLC), a prominent subtype of lung cancer, is a leading cause of cancer-related mortality [1–3]. Current treatment for NSCLC encompasses a comprehensive approach involving surgery, radiation therapy, systemic therapies, and targeted therapies [4–6]. Surgical resection remains pivotal for early-stage NSCLC, while radiation therapy, including stereotactic body radiation therapy, is crucial for locally advanced cases or inoperable tumors [1–3]. Systemic therapies, such as chemotherapy and immunotherapy, are employed based on disease stage and patient characteristics [1–3].

Targeted therapies have revolutionized NSCLC treatment by targeting specific molecular abnormalities driving tumorigenesis [7–11]. Prominent examples include tyrosine kinase inhibitors (TKIs), gefitinib, osimertinib for EGFR mutations, and crizotinib, ceritinib, alectinib for anaplastic lymphoma kinase (ALK) rearrangements [4, 9, 11, 12]. Immune checkpoint inhibitors, including pembrolizumab and nivolumab, are also emerging targeted therapies [13, 14]. These therapies have significantly improved outcomes in NSCLC by targeting specific genetic or protein-based abnormalities, and ongoing research continues to refine and expand treatment options and targets [7–11].

Mitochondrial hyperfunction has garnered increasing attention in NSCLC due to its potential influence on tumor growth [15–19]. The mitochondria, central to cellular energy metabolism, play a crucial role in tumorigenesis of NSCLC [7, 20–22]. In NSCLC, alterations in mitochondrial function, including increased oxidative phosphorylation (OXPHOS) and elevated ATP production, have been observed, contributing to the heightened bioenergetic demands of rapidly proliferating cancer cells. These hyperfunctional mitochondria provide a growth advantage by sustaining the energy-intensive processes required for tumor growth and progression [23]. Moreover, mitochondrial hyperfunction may facilitate resistance to certain therapeutic interventions, making it a compelling target for therapeutic strategies of NSCLC [15–19].

The expression and function of multiple mitochondrial proteins are altered in mitochondria of NSCLC cells, contributing to NSCLC cell growth and progression [7, 20–22]. ADCK2, or aarF domain containing kinase 2, is mitochondrial protein crucial for coenzyme Q (CoQ) biosynthesis and fatty acid metabolism [24]. We have shown that ADCK2 overexpression in NSCLC tissues correlates with poor overall survival [24]. In NSCLC cells, downregulating ADCK2 levels through shRNA or CRISPR/Cas9 KO inhibited NSCLC cell viability, proliferation, and mobility, induced apoptosis, disrupted mitochondrial functions, and downregulated Akt-mTOR signaling [21]. Depletion of ADCK2 also hindered NSCLC xenograft growth in mice [21]. YME1L (YME1 Like 1 ATPase) is a mitochondrial inner membrane protease important for mitochondrial quality control and regulation of cell growth/apoptosis [25–28]. Xia et al., demonstrated upregulation of YME1L in NSCLC tissues and cells. Silencing or knocking out YME1L inhibited NSCLC cell growth, migration, and induced apoptosis and mitochondrial dysfunction, and suppressed NSCLC xenograft growth in mice [20]. POLRMT, or RNA polymerase mitochondrial, is an enzyme found in the mitochondria responsible for transcribing mitochondrial DNA (mtDNA) necessary for mitochondrial protein synthesis and cellular energy production. It plays a pivotal role in the maintenance and regulation of mitochondrial gene expression [29–31]. Zhou et al., demonstrated that overexpressed POLRMT is required for NSCLC cell growth in vitro and in vivo [22].

NADH:ubiquinone oxidoreductase core subunit S8 (NDUFS8), a key component of the mitochondrial respiratory chain complex I, plays a pivotal role in cellular energy metabolism [32, 33]. NDUFS8 is localized within the mitochondrial inner membrane, where it functions as an essential catalytic subunit in the transfer of electrons from NADH to ubiquinone [32, 33]. NDUFS8’s role in facilitating electron transport and proton pumping is indispensable for the establishment of the mitochondrial proton gradient, a fundamental process in adenosine triphosphate (ATP) synthesis [32, 33]. Additionally, emerging evidence suggests that NDUFS8 may have broader implications beyond its canonical bioenergetic functions, potentially influencing cellular signaling pathways and contributing to various pathological conditions [32, 33]. This study provides an in-depth exploration of NDUFS8’s expression and functional significance within NSCLC, shedding light on its implications for tumorigenesis and its therapeutic relevance.

## Material and methods

### Reagents

Cell culture reagents employed in this study, encompassing serum, culture medium, rotenone, Antimycin A and antibiotics, were procured from Hyclone (Logan, UT). The anti-NDUFS8 antibody was acquired from Abcam (Cambridge, UK), while the anti-NDUFS1 antibody was provided by Cell Signaling Tech (Danvers, MA). Reagents such as puromycin, polybrene, and various other chemicals were sourced from Sigma-Aldrich Chemicals Co. (St. Louis, MO). Fluorescence dyes utilized throughout the study were reported previously [21, 34, 35].

### Cells

A549 cell line, the primary human NSCLC cells derived from three written-informed consent patients (referred to as “pNSCLC1”, “pNSCLC2”, and “pNSCLC3”) and primary lung epithelial cells originating from two distinct donors (referred to as “pEpi1” and “pEpi2”) were obtained using the reported previously [21, 34, 35]. The three stage-II lung adenocarcinoma (LUAD) patients had not received any prior anticancer therapy before undergoing surgical tumor resection. All experimental procedures were reviewed and approved by the Ethics Committee of Soochow University, ensuring compliance with the ethical guidelines outlined in the Helsinki Declaration. Cells were routinely subjected to comprehensive assessments, including mycoplasma and microbial contamination screening, short-tandem repeat (STR) profiling, and morphological evaluation.

### Human tissues

NSCLC cancer tissues and adjacent normal lung tissues from written-informed consent patients were reported previously [21]. All research protocols involving human samples were ethically approved by the Ethics Committee of Soochow University, ensuring compliance with the ethical guidelines outlined in the Helsinki Declaration.

### Immunohistochemistry (IHC)

Paraffin-embedded tissue or xenograft sections were subjected to baking, dewaxing, and hydration, and washed with 0.3% Triton X-100 in PBS (PBST) solution. To minimize any potential non-specific binding, the sections were then incubated with 5% serum in PBST for a period of 20 min. The endogenous peroxidase activity was suppressed by the application of hydrogen peroxide. The primary antibody was subsequently applied for 12 h. A biotin-labeled IgG antibody was next applied for 2 h, and diaminobenzidine (DAB) utilized for staining after washing.

### Tissue fluorescence staining

Paraffin-embedded tissue or xenograft sections were subjected to baking, dewaxing, and hydration, washed with PBST, and blocked with goat serum. The primary antibody (anti-NDUFS8) was then incubated with the tissue section for 12 h. Subsequently, the fluorescein-labeled IgG antibody (or fluorescence dyes) were introduced and incubated for 2 h. The stained tissue sections were then scrutinized utilizing a confocal microscope (Zeiss).

### Single-cell RNA sequencing data analyses

Single-cell RNA sequencing data was processed with Seurat v5.1.0 in R. Two datasets were used: an integrated lung cancer dataset from an early study [36] accessed via figshare (10.6084/m9.figshare.c.6222221.v3), and the GSE131907 dataset from Kim et al. [37] downloaded from GEO. After quality control, data was normalized (SCTransform), integrated (rPCA), and visualized (UMAP). Cell annotations were derived from the original sources.

### Quantitative Real-time PCR (qRT-PCR)

RNA extraction from cellular or tissue lysates was accomplished using TRIzol reagents, followed by reverse transcription into cDNA employing the PrimeScript RT reagent kit from Takara Bio, Japan. Subsequently, qRT-PCR was conducted following established protocols [38], utilizing *GAPDH* mRNA as an internal control. Data quantification methods were consistent with previous protocols [22], and primers for *NDUFS8* and *NDUFS1* were provided by Genechem (Shanghai, China).

### Western blotting

Cellular/tissue lysates were separated by SDS-PAGE (10–12.5%) and transferred onto PVDF (polyvinylidene difluoride) membranes. After a 45-min blockage in 5% non-fat milk, membranes were incubated overnight at 4 °C with primary antibodies. Following PBST washing, PVDF membranes were exposed to horseradish peroxidase (HRP)-secondary antibodies for 45 min at room temperature. Enhanced chemiluminescence (ECL) visualization was then executed, and the gray value of the targeted protein band was quantified using ImageJ software. Uncropped blotting images are available in Figure S1.

### shRNA or overexpression

Lentiviral constructs harboring two distinct shRNAs targeting human *NDUFS8* (shNDUFS8-S1 or shNDUFS8-S2) or the *NDUFS8* cDNA sequence were transfected into HEK-293 cells together with lentivirus envelope constructs via Lipofectamine 3000. Lentiviral particles were subsequently introduced to cultured NSCLC cells at multiplicity of infection (MOI) = 15 for 48 h. Cells were subsequently maintained in complete medium with polybrene and subjected to puromycin selection for an additional five-seven passages. Validation of NDUFS8 silencing or overexpression was confirmed at both mRNA and protein levels. For in vivo investigations, NDUFS8 shRNA sequence or the scramble control shRNA sequence (shC) was integrated into an adeno-associated virus (AAV) construct (AAV9, Genechem). The shRNA AAV was generated through transfection of the construct into HEK-293 cells with AAV envelope constructs.

### CRISPR/Cas9-mediated knockout of NDUFS8

NSCLC cells were maintained in complete medium with polybrene and subsequently transfected with lentiviral particles containing the Cas9-expressing construct (from Dr. Cao’s group [39, 40]). Stable Cas9-expressing cells were established following puromycin selection. The small-guide (sgRNA) sequence targeting human *NDUFS8* was introduced into a lenti-CRISPR/Cas9-KO-puro construct (from Dr. Cao [39, 40]). Lentiviral particles were transfected into Cas9-expressing stable cells, and stable cells selected using puromycin. NDUFS8 KO screening, both at the DNA and protein levels, was carried out, leading to the establishment of single stable KO cells (“koNDUFS8”). Control cells were transduced with the Cas9-expressing construct with the lenti-CRISPR/Cas9-KO-puro construct (“koC”).

### Akt1 mutation

Lentiviral particles containing the S473D constitutively-active mutant Akt1 (caAkt1) (provided by Dr. Cao [41–43]) were introduced into cultured NSCLC cells. Following selection with puromycin, stable NSCLC cells expressing caAkt1 were established.

### Cellar fluorescence staining

Cells were seeded into 24-well plates at a concentration of 2 × 10^4^ cells in 600 μL of medium per well and then incubated for the designated time. Following incubation, the cells were fixed with 4% paraformaldehyde, washed with PBS, and subsequently subjected to incubation with 0.3% Triton at room temperature. The cells were then exposed to fluorescence dyes, washed, and observed under a Leica microscope. The fluorescence intensity was quantified using fluorescence spectrophotometer (Hitachi F-7000).

### Assessment of mitochondrial complex I activity and ATP levels

The enzymatic activity of mitochondrial complex I was assessed with a commercially available kit from Sigma, employing spectrophotometric techniques to monitor the conversion of NADH to NAD+ catalyzed by complex I. The reduction in absorbance at 425 nm served as a direct indicator of complex I activity. Cellular and tissue ATP levels were determined using a commercial colorimetric kit also from Sigma, following the provided protocols. Each treatment involved the assessment of 25 μL of cellular or tissue lysates.

### Measurement of reduced glutathione (GSH) to oxidized glutathione (GSSG) ratio

The quantification of the ratio between reduced glutathione (GSH) and oxidized glutathione (GSSG) was conducted using a GSH/GSSG ratio kit procured from Thermo Fisher Scientific (Suzhou, China). Lysates were treated with 5,5’-Dithio-bis (2-nitrobenzoic acid) (DTNB), glutathione reductase, and NADPH in a reaction solution, and the absorbance at 430 nm was recorded over a five-minute interval employing a spectrophotometer. A standard curve was established using GSH and GSSG standards to quantify their concentrations within the lysates.

### Thiobarbituric acid reactive substances (TBAR) assay

A TBAR assay kit was employed. Tissue or cellular protein lysates were allowed to react with thiobarbituric acid (TBA) to form the TBAR complex. Following this reaction, and after cooling and centrifugation to eliminate any precipitate, the absorbance at 535 nm was determined using a spectrophotometer.

### CCK8 assay

NSCLC cells or lung epithelial cells subjected to genetic modifications were seeded at a density of 4000 cells per well in 96-well plates. Subsequently, cells were incubated in a 37 °C, 5% CO_2_ incubator for an additional 72 h. The CCK8 mixture was then added and incubated for an additional 2 h, followed by measurement of CCK8 absorbance at 450 nm using a microplate reader.

### Caspase activity

Caspase-3/-9 activity in the described cell lysates was assessed using a Caspase-3/-9 colorimetric assay kit (BioVision, Milpitas, CA) in accordance with the manufacturer’s instructions.

### Transwell assays

NSCLC cells with genetic modifications were suspended at a density of 1.5 × 10^4^ cells per well in serum-free medium and added to the upper surface of “Transwell” chambers. After 24 h, migrated cells on the lower surface were fixed with 70% ice ethanol, stained, and photographed. For in vitro cell invasion assays, the chamber were always coated with Matrigel (Sigma), with the other procedures remained consistent.

### Flow cytometry

NSCLC cells subjected to genetic modifications/treatments were centrifuged, resuspended, and stained with Annexin V and/or propidium iodide (PI) (Keji biology, Nanjing, China) in the binding buffer. Flow cytometry was employed to assess cell apoptosis or cell cycle progression.

### Cell death assay

Cells were plated into six-well plates (at 1 × 10^5^ per well). Following the designated treatment, cell death was tested by Trypan blue staining using an automatic cell counter.

### mTOR kinase activity assay

NSCLC cells, with designated NDUFS8 genetic modification, were further constructed to stably express FLAG-tagged mTOR (Genechem, Shanghai, China). Cells were then lysed in ice-cold CHAPS lysis buffer [44]. Clarified lysates were immunoprecipitated with anti-FLAG agarose beads for 2 h at 4 °C [44]. Beads were pelleted (500 × *g*, 30 s), washed thrice with ice-cold buffer [44], and resuspended in mTOR kinase assay buffer containing 750 µM ATP to initiate phosphorylation of GST-tagged p70S6K1. Reactions were terminated after 15 min [44], and phospho-p70S6K (Thr389) levels were quantified via anti-phospho-p70S6K antibody, HRP-conjugated secondary antibody, and TMB (3,3’,5,5’-tetramethylbenzidine) substrate. The absorbance (450 nm) was measured, and kinase activity was normalized to control samples.

### Xenograft studies

Xenograft experiments were conducted using 5–6-week-old nude mice, consisting of an equal distribution of male and female individuals with a weight range of 18.4–19.0 g. These mice were housed in the Animal Facility at Soochow University. Five million pNSCLC1 cells per mouse were subcutaneously (*s.c*.) injected into the flanks. Tumor growth was monitored, and once each xenograft reached a volume of 100 mm^3^, about three weeks post-injection, the pNSCLC1 xenograft-bearing nude mice were randomly assigned to two groups. These groups received intratumoral injections of the specified adeno-associated virus (aav) at a volume of 1.8 μL per xenograft, with a viral titer of 1.2 × 10^9^ plaque-forming units (PFU) per injection. The virus was injected twice. Tumor dimensions were measured and volumes were calculated using a previously described formula [21, 34, 45]. Ethical approval for all animal experiments was obtained from the Institutional Animal Care and Use Committee (IACUC) and the Ethics Board of Soochow University.

### Statistical analyses

A blinded approach was utilized for allocating groups for in vitro studies. These experiments were replicated across five distinct biological repetitions. Data displaying a normal distribution were presented as mean ± standard deviation (SD). Statistical analysis was conducted using SPSS version 22.0 (SPSS Co., Chicago, IL). The unpaired Student’s *t*-test was employed for comparisons between two specific groups, while one-way ANOVA with the Scheffe’ and Tukey Test was utilized for comparisons involving more than two groups. Statistical significance was established when *P*-values were less than 0.05.

## Results

### Overexpression of *NDUFS8* correlates with poor prognosis of NSCLC

**Fig. 1:**
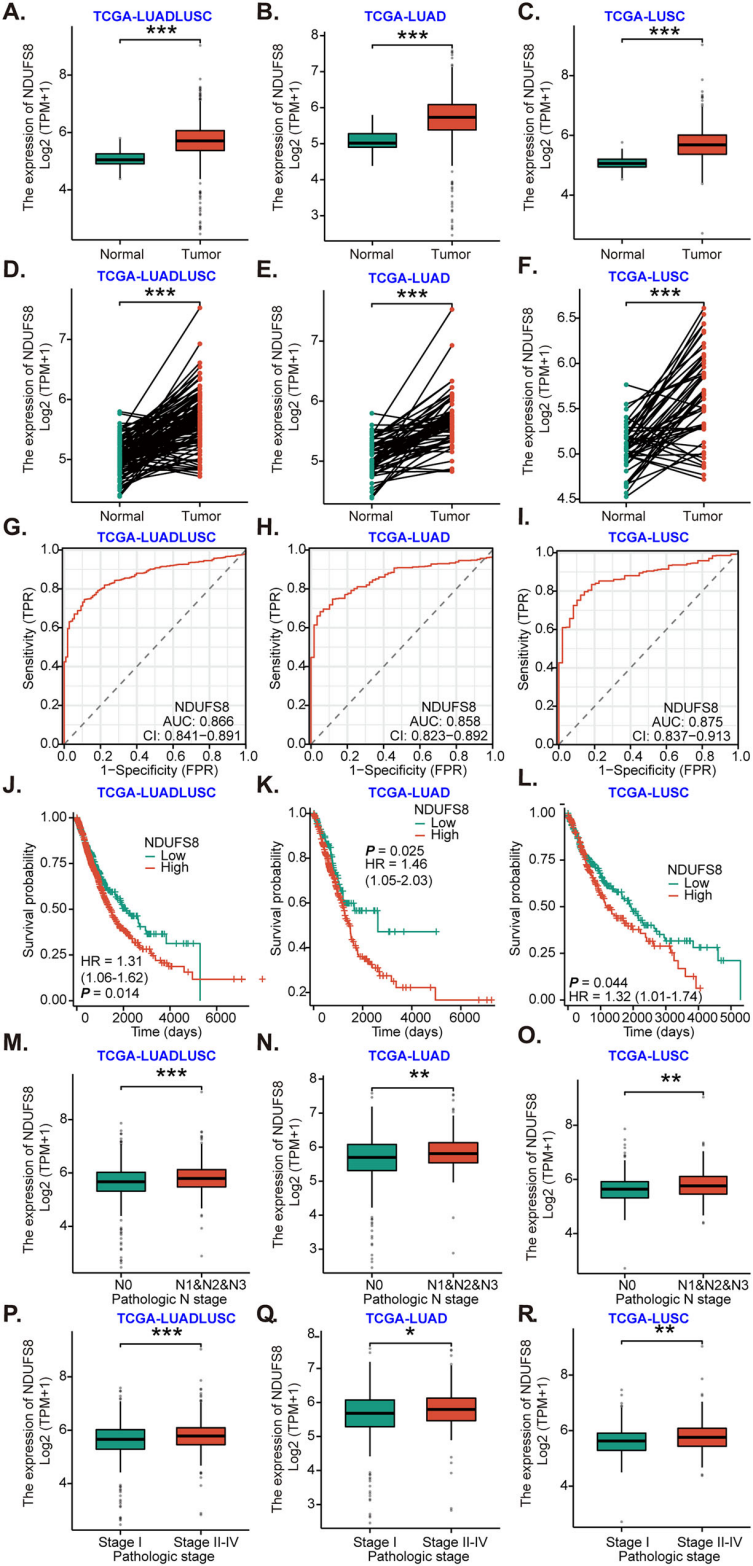
Overexpression of *NDUFS8* correlates with poor prognosis of NSCLC. The Cancer Genome Atlas (TCGA) lung adenocarcinoma (LUAD) lung squamous cell carcinoma (LUSC) cohort (combining both LUAD and LUSC), TCGA-LUAD cohort and TCGA-LUSC cohort show *NDUFS8* expression (RNA-Seq) in NSCLC tissues (“Tumor”), normal lung tissues (“Normal”) (**A**–**C**) and also in paired cancer-surrounding normal tissues (**D**–**F**). The receiver operating characteristic (ROC) curve results demonstrated the relationship between *NDUFS8* overexpression and the potential predictive effect on NSCLC patients’ survival (**G**–**I**). The Kaplan-Meier survival analyses show that the association between *NDUFS8* expression and the overall survival of NSCLC patients (**J**–**L**) and subgroup NSCLC patients (**M**–**R**). Transcripts Per Million (TPM), True Positive Rate (TPR) and False Positive Rate (FPR). *P < 0.05. **P < 0.01. ***P < 0.001.

We first conducted an analysis utilizing data from The Cancer Genome Atlas (TCGA) program to investigate the expression of *NDUFS8* in NSCLC. NSCLC comprises two primary subtypes: lung adenocarcinoma (LUAD) and lung squamous cell carcinoma (LUSC). Our examination of the TCGA LUADLUSC dataset (including both LUAD and LUSC) revealed that the quantity of *NDUFS8* transcripts in cancerous tissues (“Tumor”) was significantly higher than in normal lung tissues (“Normal”) (Fig. 1A). Additionally, the expression of *NDUFS8* in LUAD (Fig. 1B) or LUSC (Fig. 1C) cancer tissues was significantly elevated in comparison to normal lung tissues. When we performed a paired tissue analysis, the expression of *NDUFS8* in LUADLUSC cancer tissues (“Tumor”) was notably higher than in the corresponding adjacent normal tissues (“Normal”) (Fig. 1D). Furthermore, in LUAD (Fig. 1E) or LUSC (Fig. 1F) cancer tissues, the number of *NDUFS8* transcripts was also higher than in their matched surrounding normal tissues. This comprehensive bioinformatics investigation affirms that *NDUFS8* expression is upregulated in NSCLC.

The Area Under the Curve (AUC) serves as an effective method to condense and predict the overall diagnostic accuracy of a specific molecule in human cancer. ROC curve was then employed to assess the potential diagnostic utility of *NDUFS8* expression in NSCLC. With an AUC of 0.866, in Fig. 1G, overexpression of *NDUFS8* demonstrates a significant value in predicting potential unfavorable survival outcomes in LUADLUSC patients. Furthermore, *NDUFS8* overexpression also demonstrates substantial predictive value for poor prognosis in LUAD (Fig. 1H) or LUSC (Fig. 1I) patients.

Of great significance, we observed that *NDUFS8* overexpression is associated with an unfavorable prognosis and poor survival in LUADLUSC patients (Fig. 1J). Additionally, higher levels of *NDUFS8* expression in LUAD (Fig. 1K) or LUSC (Fig. 1L) were correlated with poorer patient prognoses. Subgroup analysis, taking into consideration different clinical characteristics, revealed that *NDUFS8* overexpression is significantly linked with higher N-stage in LUADLUSC patients (Fig. 1M), as well as in LUAD (Fig. 1N) or LUSC patients (Fig. 1O). Furthermore, *NDUFS8* expression in pathological advanced stages (Stage II-IV) of LUADLUSC is higher than that in the Stage-I LUADLUSC (Fig. 1P). *NDUFS8* overexpression is also associated with higher stages in LUAD (Fig. 1Q) or LUSC (Fig. 1R) patients. NDUFS6, another important component of the NADH:ubiquinone oxidoreductase complex, shares functional similarities with NDUFS8 [46]. While the analyses of TCGA cohort demonstrate upregulated *NDUFS6* expression in NSCLC (Fig. S2A), this overexpression does not exhibit a significant correlation with patient prognosis (Fig. S2B). These findings collectively establish that *NDUFS8* is consistently overexpressed in NSCLC and is strongly correlated with poor overall survival, elevated N stages, and advanced pathological stages.

### NDUFS8 is overexpressed in local NSCLC tissues and various NSCLC cells

**Fig. 2:**
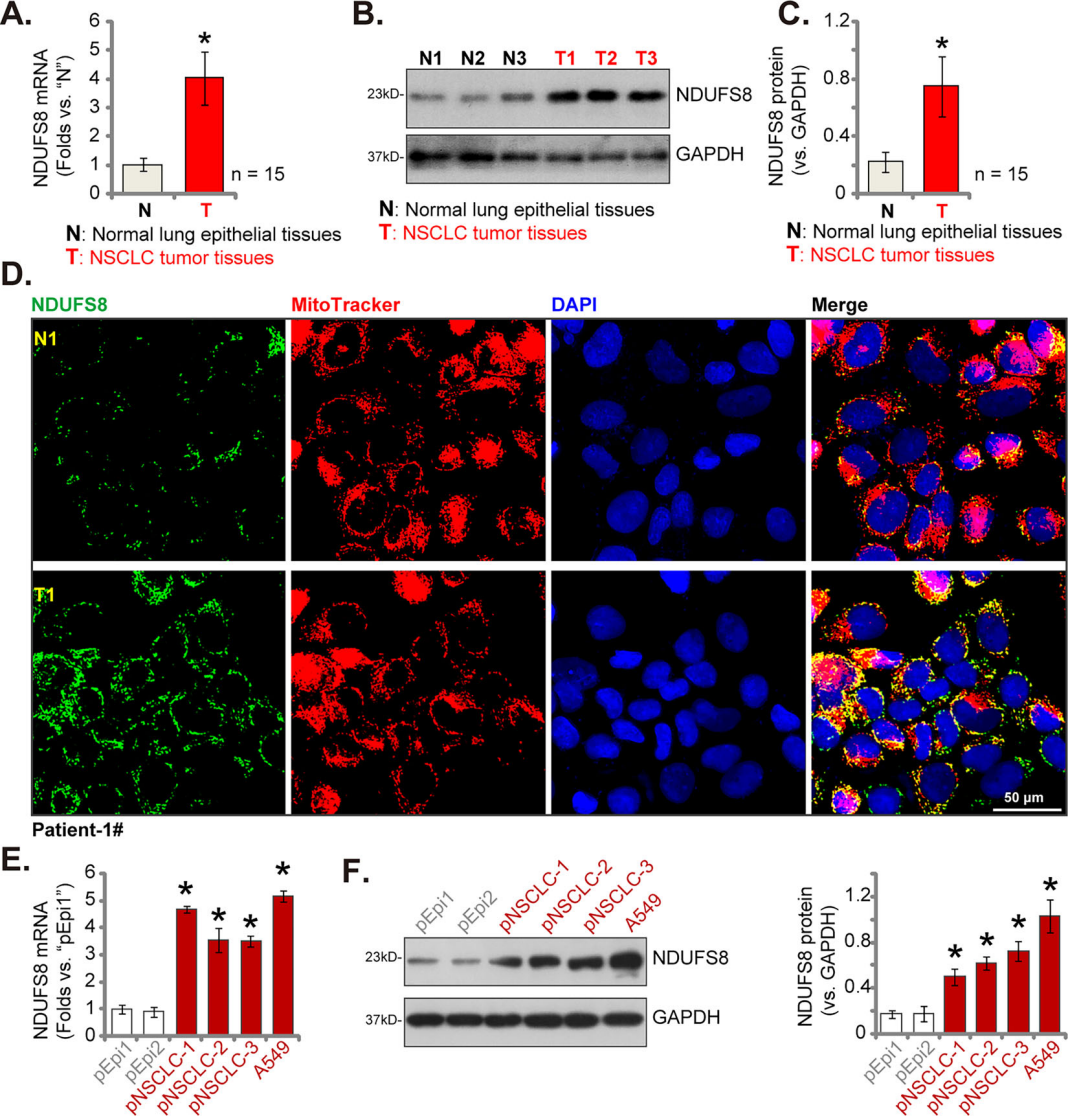
NDUFS8 is overexpressed in local NSCLC tissues and various NSCLC cells. The expression profiles of NDUFS8 at both the mRNA and protein levels were examined in NSCLC tumor tissues (“T”) and their corresponding adjacent normal lung epithelial tissues (“N”) obtained from a cohort of fifteen primary NSCLC patients (*n* = 15) (**A**–**C**). Tissue immunohistofluorescence images were employed to illustrate the subcellular localization of NDUFS8 (in green), MitoTracker (in red), and nuclear marker DAPI (in blue) within both the NSCLC tumor tissue slide and the adjacent normal lung epithelial tissue of “Patient-1#” (**D**). *NDUFS8* mRNA and protein expression in described NSCLC cells and lung epithelial cells was shown (**E**, **F**). Error bars in the figures represent the mean ± standard deviation (SD), with statistical significance (*P < 0.05) when comparing “N” tissues or “pEpi1” cells. Scale bar = 50 μm.

We next tested the expression profile of NDUFS8 within localized NSCLC tissues. Our study comprised fifteen (*n* = 15) primary NSCLC patients diagnosed with LUAD at stage III-IV as reported previously [22]. The analysis focused on a comparative assessment of NDUFS8 expression in NSCLC tumor tissues (“T”) and adjacent normal lung epithelial tissues (“N”). A striking upregulation of *NDUFS8* mRNA expression in NSCLC tissues was detected when contrasted with its expression within normal lung epithelial counterparts (Fig. 2A). Furthermore, Western blotting analyses unveiled a notable increase in NDUFS8 protein levels within NSCLC tumor tissues from three representative patients, denoted as “T1” to “T3” (Fig. 2B). An extensive evaluation of NDUFS8 protein blotting data across all 15 tissue sets supported a significant upregulation in NDUFS8 protein within NSCLC tumor tissues (Fig. 2C).

To elucidate the subcellular localization of NDUFS8, we conducted tissue immunohistofluorescence assays employing a previously established protocol [41, 47]. As displayed in Fig. 2D, NDUFS8 protein, represented by green fluorescence, exhibited a clear co-localization with the mitochondrial marker MitoTracker (visualized in red fluorescence [41, 47]) in both the NSCLC tumor section (“T1”) and the corresponding adjacent normal lung epithelial section (“N1”) from a representative patient, identified as “Patient-1#.” Notably, the green fluorescence intensity of NDUFS8 in NSCLC tumor slides was substantially higher than that observed in adjacent lung epithelial tissues (Fig. 2D).

Subsequent investigations centered on the assessment of NDUFS8 expression in diverse NSCLC cells, encompassing primary human NSCLC cells (“pNSCLC1/2/3”, derived from three different patients [20, 45, 48]) and immortalized A549 cells. Our findings revealed a significant elevation in *NDUFS8* mRNA expression within both primary and immortalized NSCLC cells, in stark contrast to primary human lung epithelial cells (“pEpi1” and “pEpi2,” derived from two donors [20]) (Fig. 2E). Moreover, the upregulation of NDUFS8 protein levels was consistently observed across various NSCLC cells, while its expression remained substantially lower in lung epithelial cells (Fig. 2F). Collectively, these results underscore the robust overexpression of NDUFS8 within localized NSCLC tissues and diverse NSCLC cells, shedding light on the potential significance of NDUFS8 in the progression of NSCLC.

### Single-cell RNA sequencing reveals upregulation of *NDUFS8* in cancerous cells of NSCLC

**Fig. 3:**
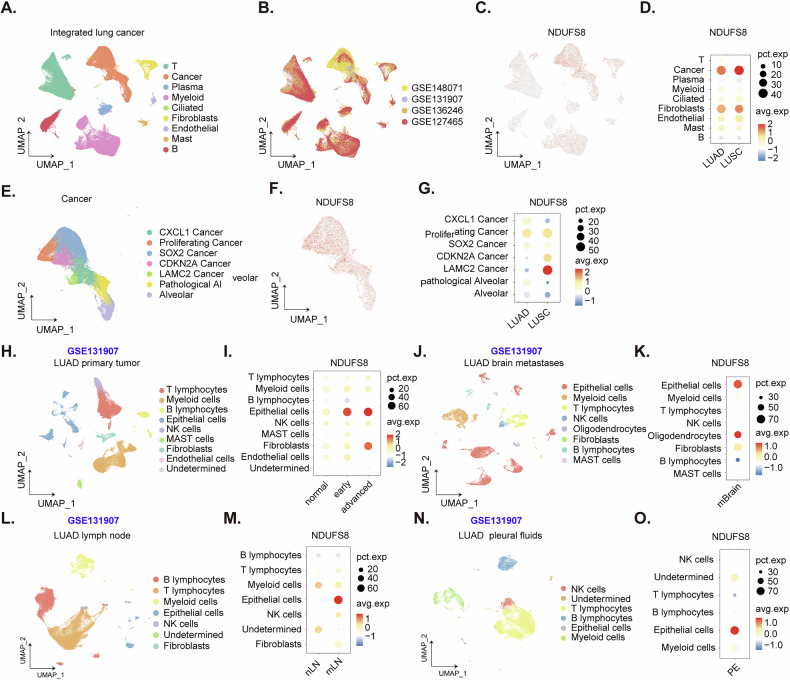
Single-cell RNA sequencing reveals upregulation of *NDUFS8* in cancerous cells of NSCLC. Dimensionality reduction plots showing annotated cell populations from integrated scRNA-seq data (**A**). Source integration plots indicating dataset origins (**B**). Dot plots (**C**) and expression density plots (**D**) illustrate high *NDUFS8* expression in cancer cells and fibroblasts in both LUAD and LUSC. Clustering analyses of NSCLC cancer cells (**E**–**G**) reveal sustained *NDUFS8* expression, especially in the LAMC2 cancer cell group (**G**). NDUFS8 expression in epithelial and fibroblast populations in primary LUAD, increasing with cancer progression (**H**, **I**). Elevated *NDUFS8* expression in brain metastases (**J**, **K**), and in metastatic lymph nodes (**L**, **M**). Upregulation of *NDUFS8* in LUAD pleural effusion samples (**N**, **O**).

We analyzed the integrated single-cell RNA sequencing (scRNA-seq) data for lung cancer, which were derived from the integrated lung cancer dataset by Prazanowska et al. [36] and shared on figshare (10.6084/m9.figshare.c.6222221.v3). We downloaded the Seurat object, which includes cell annotations, and accessed the GSE148071, GSE131907, GSE136246, and GSE127465 datasets within this object using the Seurat package in R. The data revealed critical insights into the expression of *NDUFS8* across various cell populations. The dimensionality reduction plots clearly illustrated the cell annotations (Fig. 3A) and the sources of the integrated data (Fig. 3B), facilitating a comprehensive understanding of the cellular composition. Subsequent dot plots (Fig. 3C) and expression density plots (Fig. 3D) demonstrated that *NDUFS8* is predominantly expressed in cancer cells and fibroblasts. Elevated levels of *NDUFS8* expression were observed in cancer cell populations from both LUAD and LUSC (Fig. 3C, D), indicating its relevance across different lung cancer subtypes. Further analysis involved the extraction and clustering of cancer cell populations (Fig. 3E–G). Within these clusters, *NDUFS8* maintained high expression levels, with particularly pronounced expression in the LAMC2 cancer cell group (Fig. 3G). These findings emphasizes the potential significance of NDUFS8 in specific cancer cell subtypes and its role in NSCLC biology.

Next, we analyzed the scRNA-seq data from the study by Kim et al. [37], specifically the GSE131907 dataset. We downloaded the expression matrix and cell annotation files from the GEO website (https://www.ncbi.nlm.nih.gov/geo/query/acc.cgi?acc=GSE131907) and performed standard dimensionality reduction using the Seurat package. Cellular annotations provided by the original authors [37] facilitated accurate identification of cell populations. In primary LUAD tumors, *NDUFS8* was expressed in both epithelial and fibroblast cell populations (Fig. 3H, I). Moreover, its expression increased with cancer progression, suggesting its potential role in tumorigenesis and cancer progression.Within LUAD brain metastases, *NDUFS8* expression was significantly elevated in epithelial cells, oligodendrocytes, and fibroblasts (Fig. 3J, K). This suggests its possible involvement in metastatic adaptation and survival within the unique brain microenvironment. Note that the presence of oligodendrocytes is likely due to the inclusion of brain metastasis samples in this dataset (Fig. 3J, K). In metastatic lymph nodes, *NDUFS8* was highly expressed in epithelial cells (Fig. 3L, M). Additionally, *NDUFS8* was upregulated in epithelial cells within LUAD pleural effusion samples, further emphasizing its role in LUAD progression (Fig. 3N, O). Collectively, these findings demonstrate the widespread upregulation of *NDUFS8* in cancerous cells of LUAD, suggesting its significance as a biomarker for disease progression and metastasis. Further investigation into the functional role of *NDUFS8* in cancer biology is warranted.

### NDUFS8 silencing disrupts mitochondrial functions in NSCLC cells

**Fig. 4:**
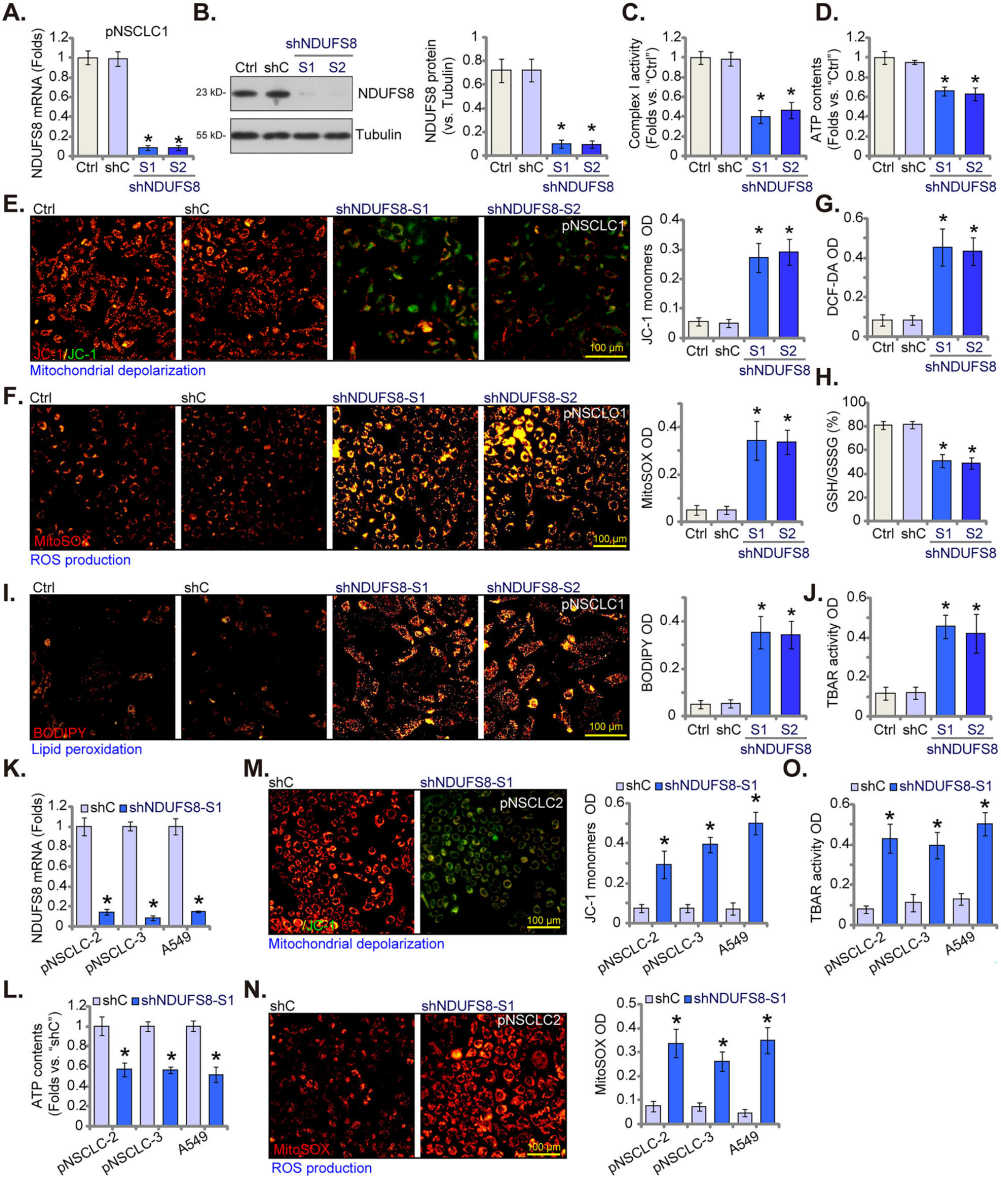
NDUFS8 shRNA disrupts mitochondrial functions in NSCLC cells. The primary human NSCLC cells, pNSCLC1, were engineered to stably express lentivirus-packed NDUFS8-targeting shRNA (shNDUFS8-S1 or shNDUFS8-S2), or a scramble control shRNA (“shC”). Following a 24-h cultivation period, *NDUFS8* mRNA (**A**) and protein (**B**) expression was measured. The mitochondrial complex I activity (**C**) and cellular ATP contents (**D**) were measured. Mitochondrial depolarization was evaluated based on JC-1 monomer intensity (**E**), while ROS production was measured by MitoSOX and DCF-DA dyes (**F**, **G**). Furthermore, the GSH to GSSG ratio (**H**) and lipid peroxidation levels, as indicated by BODIPY intensity (**I**) and TBAR activity (**J**), were determined. Additional primary human NSCLC cells, namely pNSCLC2 and pNSCLC3, as well as immortalized A549 cells, were engineered to maintain stable expression of lentivirus-delivered shNDUFS8-S1 or shC. Following a 24 h cultivation period, *NDUFS8* mRNA (**K**) was measured. ATP contents (**L**), depolarization of mitochondria (JC-1 monomer intensity, **M**), ROS production (MitoSOX intensity, **N**) and lipid peroxidation (TBAR activity, **O**) were tested similarly. “Ctrl” stands for the parental control cells. Error bars in the figures represent the mean ± standard deviation (SD), with statistical significance (* *P* < 0.05) when comparing “shC” cells. The experiments depicted in this figure were independently repeated five times (*n* = 5) and consistently yielded similar results. Scale bar =100 μm.

In order to explore the potential function of NDUFS8 in NSCLC cells, shRNA method was utilized to silence NDUFS8. Specifically, the primary human NSCLC cells, pNSCLC1, were stably transduced to lentivirus-packed shRNA targeting NDUFS8: shNDUFS8-S1 or shNDUFS8-S2 (containing non-overlapping shRNA sequences). As compared to the control pNSCLC1 cells with scramble control shRNA (“shC”), *NDUFS8* mRNA (Fig. 4A) and protein (Fig. 4B) expression was substantially decreased in shNDUFS8-expressing cells. NDUFS8 silencing disrupted mitochondrial functions, inhibiting mitochondrial complex I activity (Fig. 4C) and decreased cellular ATP contents (Fig. 4D) in pNSCLC1 cells. Mitochondrial depolarization was detected in shNDUFS8-expressing pNSCLC1 cells, supported by JC-1 conversion of red fluorescent aggregates to green fluorescent monomers (Fig. 4E). Significant reactive oxygen species (ROS) production was also detected in NDUFS8-silenced pNSCLC1 cells. The MitoSOX fluorescence intensity was significantly increased in pNSCLC1 cells with NDUFS8 shRNAs (Fig. 4F). Moreover, NDUFS8 silencing increased DCF-DA green fluorescence intensity (Fig. 4G), whiling decreasing GSH/GSSG ratio (Fig. 4H) in pNSCLC1 cells, again supporting ROS production and oxidative injury. Further experimental results showed that NDUFS8 shRNA caused lipid peroxidation in pNSCLC1 cells. BODIPY lipid droplet dyes can localize the polar lipids in the cell to specifically stain the lipid droplets. The BODIPY dye intensity was significantly enhanced in shNDUFS8-S1/2-expressiong pNSCLC1 cells (Fig. 4I). Moreover, TBAR assay results showed increased malondialdehyde (MDA) contents in NDUFS8-silenced pNSCLC1 cells (Fig. 4J), further supporting lipid peroxidation.

The potential effect of NDUFS8 on other NSCLC cells was also tested. The primary human NSCLC cells derived from two other patients, pNSCLC2 and pNSCLC3, as well as the immortalized A549 cells were infected with shNDUFS8-S1-expressing lentiviral particles and stable cells formed with puromycin-based selection. When compared to control cells with shC, *NDUFS8* mRNA expression was significantly decreased in the shNDUFS8-S1-expressing NSCLC cells (Fig. 4K). NDUFS8 silencing decreased ATP contents in the primary and immortalized NSCLC cells (Fig. 4L). Moreover, mitochondrial depolarization, evidenced by JC-1 green monomers accumulation (Fig. 4M), and ROS production, tested by MitoSOX intensity increasing (Fig. 4N), were observed in shNDUFS8-S1-expressing NSCLC cells. Moreover, TBAR assay results showed increased MDA contents in shNDUFS8-S1-expressing NSCLC cells, supporting lipid peroxidation (Fig. 4O). These results showed that NDUFS8 shRNA disrupted mitochondrial functions and induced ATP depletion, ROS production and lipid peroxidation in NSCLC cells.

### NDUFS8 silencing provokes apoptosis activation in NSCLC cells

**Fig. 5:**
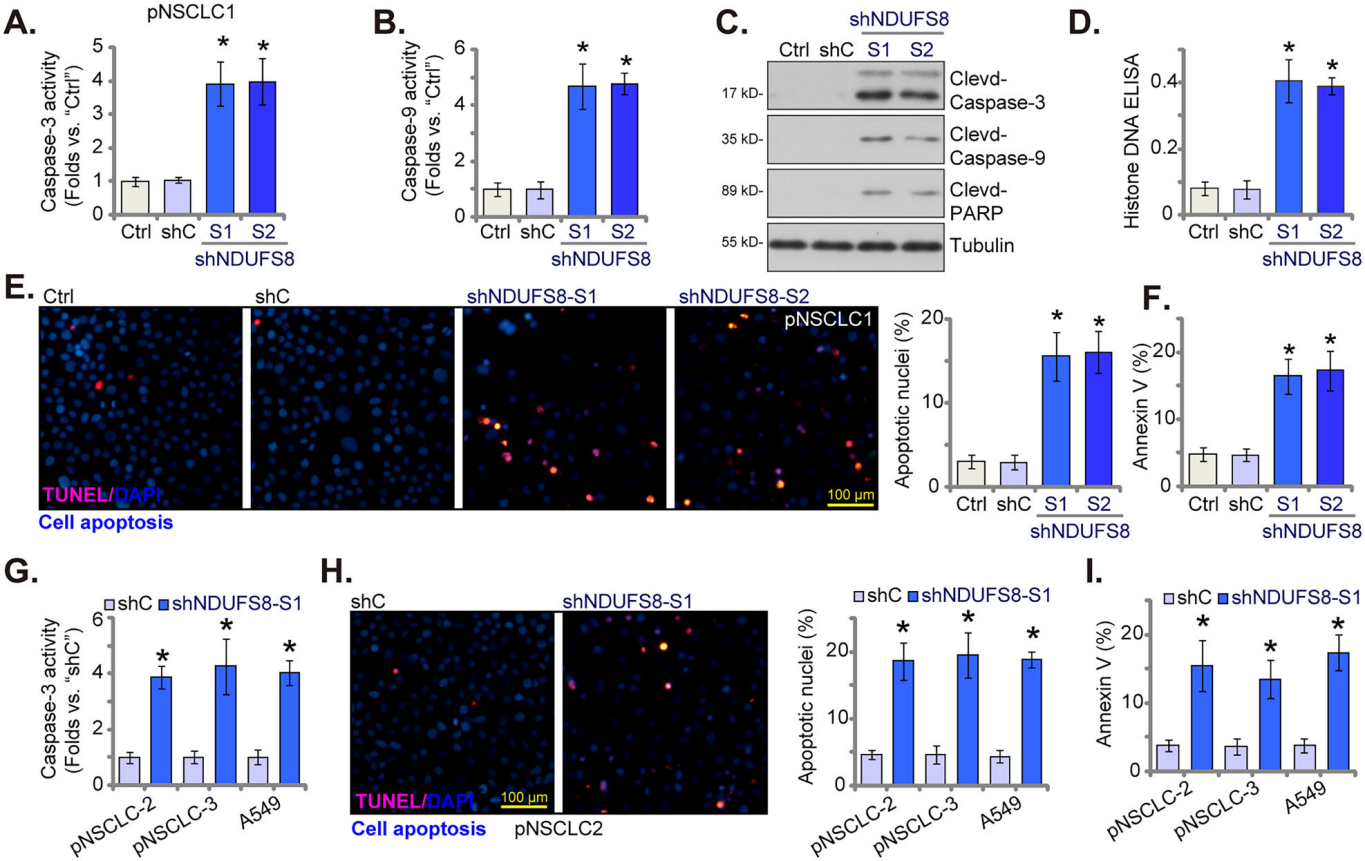
NDUFS8 silencing provokes apoptosis in NSCLC cells. The primary human NSCLC cells, pNSCLC1, were engineered to stably express lentivirus-packed NDUFS8-targeting shRNA (shNDUFS8-S1 or shNDUFS8-S2), or a scramble control shRNA (“shC”). Following a 60-h cultivation period, the Caspase-3 activity (**A**), the Caspase-9 activity (**B**), expression of apoptosis-associated proteins (**C**) and Histone-bound DNA contents (**D**) were tested. Cell apoptosis was examined via nuclear TUNEL staining (**E**) and Annexin V FACS (**F**) assays, with results quantified. Additional primary human NSCLC cells, namely pNSCLC2 and pNSCLC3, as well as immortalized A549 cells were engineered to maintain stable expression of lentivirus-delivered shNDUFS8-S1 or shC. Following a 60-h cultivation period, the Caspase-3 activity was tested (**G**). Cell apoptosis was similarly examined via nuclear TUNEL staining (**H**) and Annexin V FACS (**I**) assays. “Ctrl” stands for the parental control cells. Error bars in the figures represent the mean ± standard deviation (SD), with statistical significance (* *P* < 0.05) when comparing “shC” cells. The experiments depicted in this figure were independently repeated five times (*n* = 5) and consistently yielded similar results. Scale bar = 100 μm.

Mitochondrial disruption can provoke apoptosis cascade [49]. We therefore tested whether silencing of NDUFS8 could provoke apoptosis in NSCLC cells. In pNSCLC1 cells, silencing of NDUFS8, by shNDUFS8-S1 or shNDUFS8-S2, increased the activity of Caspase-3 (Fig. 5A) and Caspase-9 (Fig. 5B). Moreover, NDUFS8 shRNA induced cleavages of Caspase-3, Caspase-9 and poly (ADP-ribose) polymerase 1 (PARP1) in pNSCLC1 cells (Fig. 5C). The Histone-bound DNA contents, tested by ELISA assays, were increased in shNDUFS8-expressing pNSCLC1 cells (Fig. 5D), supporting increased DNA breaks. NDUFS8 shRNA induced apoptosis activation in pNSCLC-1 cells, evidenced by increased TUNEL-positive nuclei ratio (Fig. 5E). Furthermore, the increased number of Annexin V-stained pNSCLC-1 cells after NDUFS8 silencing again supported apoptosis activation (Fig. 5F). In other primary cells, pNSCLC2 and pNSCLC3, and also in immortalized A549 cells, shNDUFS8-S1-induced silencing of NDUFS8 increased Caspase-3 activity (Fig. 5G). Moreover, apoptosis activation was induced, as the TUNEL-positive nuclei ratio (Fig. 5H) and Annexin V-positive cell percentage (Fig. 5I) were both significantly increased in shNDUFS8-S1-treated cells. Therefore, silencing of NDUFS8 provoked apoptosis in NSCLC cells.

### NDUFS8 silencing inhibits NSCLC cell growth, cell cycle progression, migration and invasion

**Fig. 6:**
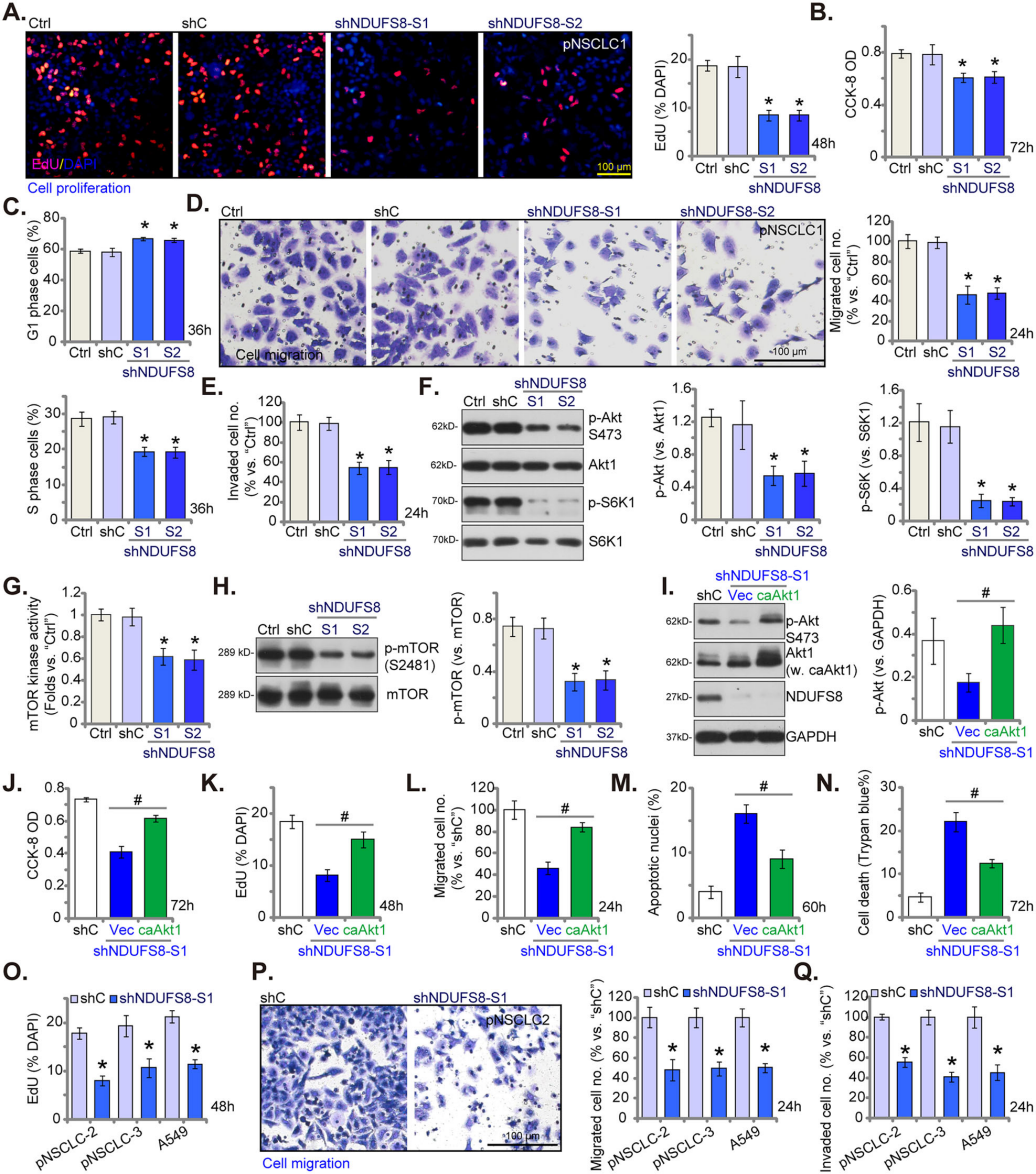
NDUFS8 silencing inhibits NSCLC cell growth, cell cycle progression, migration and invasion. The primary human NSCLC cells, pNSCLC1, were engineered to stably express lentivirus-packed NDUFS8-targeting shRNA (shNDUFS8-S1 or shNDUFS8-S2), or a scramble control shRNA (“shC”). Following cultivation of indicated time periods, cell proliferation, viability and cell cycle progression were tested via nuclear EdU staining (**A**), CCK-8 (**B**) and PI-FACS assays (**C**), respectively. Cell migration (**D**) and invasion (**E**) were measured via “Transwell”-based assays. Expression of listed proteins was tested via Western blotting assays (**F**, **H**). The mTOR kinase activity was also measured (**G**). The pNSCLC-1 cells expressing shNDUFS8-S1 were further transduced with a constitutively active Akt1 (“caAkt1”, Ser473) construct or the empty vector (“Vec”), control cells were with shC, and expression of listed proteins was shown (**I**). These cells were further cultured for designated time, cell vitality, proliferation, migration, apoptosis and death were assessed via CCK-8 (**J**), nuclear EdU incorporation (**K**), “Transwell” (**L**), nuclear TUNEL staining (**M**) and Trypan blue exclusion (**N**) assays respectively, with results quantified. Additional primary human NSCLC cells, pNSCLC2 and pNSCLC3, as well as immortalized A549 cells were engineered to maintain stable expression of lentivirus-delivered shNDUFS8-S1 or shC. Following cultivation of indicated time periods, cell proliferation (**O**), migration (**P**) and invasion (**Q**) were tested similarly. “Ctrl” stands for the parental control cells. Error bars in the figures represent the mean ± standard deviation (SD), with statistical significance (* *P* < 0.05) when comparing “shC” cells. ^**#**^ *P* < 0.05 (**I**–**N**). The experiments depicted in this figure were independently repeated five times (*n* = 5) and consistently yielded similar results. Scale bar = 100 μm.

The overactivity of mitochondria has emerged as a significant contributor to the development and progression of NSCLC cells [7, 20–22]. Hence, we studied the potential impact of silencing NDUFS8 on the progression of NSCLC cells in vitro. In primary pNSCLC1 cells, silencing of NDUFS8 using shNDUFS8-S1 or shNDUFS8-S2 resulted in the inhibition of cell growth and a decrease in the proportion of EdU-positive nuclei (Fig. 6A). Cell viability, tested by the CCK-8 assay, was also decreased (Fig. 6B). NDUFS8 silencing also disrupted the cell cycle, leading to G1-S phase arrest in pNSCLC1 cells (Fig. 6C). There was a significant increase in the percentage of G1-phase cells, along with a marked decrease in the percentage of S-phase cells (Fig. 6C). The application of NDUFS8 shRNAs also robustly inhibited the in vitro migration (Fig. 6D) and invasion (Fig. 6E) of pNSCLC1 cells, which were evaluated through “Transwell” and “Matrigel Transwell” assays, respectively.

Previous investigations have demonstrated that mitochondrial hyperactivity is important for the Akt-mTOR signaling pathway activation in NSCLC cells [20, 21]. Consequently, we investigated the potential influence of NDUFS8 on this signaling cascade. In pNSCLC-1 cells, silencing NDUFS8 by shNDUFS8-S1/2 significantly inhibited the phosphorylation of Akt and S6K1 (Fig. 6F). ATP is required for mTOR kinase activity, as it serves as the phosphate donor during the catalytic phosphorylation of substrates [50–52]. This includes the phosphorylation of Akt and S6K [50–52]. The ATP-dependent activity of mTOR underscores its role in coupling cellular energy status to growth and metabolic regulation [50–52]. We showed that the mTOR kinase activity (Fig. 6G) as well as mTOR phosphorylation at Ser-2481 (Fig. 6H) were both decreased in NDUFS8-silenced pNSCLC-1 cells. The total protein expression of Akt1, S6K1 and mTOR remained largely unaltered (Fig. 6F, H). Subsequently, we transduced pNSCLC-1 cells expressing shNDUFS8-S1 with a constitutively active Akt1 (caAkt1, Ser473), which successfully restored Akt activation without affecting NDUFS8 expression (Fig. 6I). Importantly, the reduction in cell viability (CCK-8 OD) (Fig. 6J), inhibition of proliferation (Fig. 6K), and suppression of migration (Fig. 6L) induced by shNDUFS8-S1 were partially ameliorated by the introduction of caAkt1. Furthermore, restoring Akt activation through caAkt1 also attenuated shNDUFS8-S1-induced apoptosis (Fig. 6M) and cell death (Fig. 6N) in pNSCLC-1 cells. These findings demonstrate that NDUFS8 exerts its effects on NSCLC cells, at least in part, through the regulation of the Akt signaling pathway.

In other primary cells, pNSCLC2 and pNSCLC3, as well as immortalized A549 cells, silencing NDUFS8 with shNDUFS8-S1 similarly hindered cell proliferation and reduced the ratio of EdU-positive nuclei (Fig. 6O). Additionally, NDUFS8 silencing had a suppressive effect on in vitro cell migration (Fig. 6P) and invasion (Fig. 6Q). Thus, NDUFS8 plays a crucial role in the progression of NSCLC cells.

### NDUFS8 knockout exerts significant anti-NSCLC cell activity

**Fig. 7:**
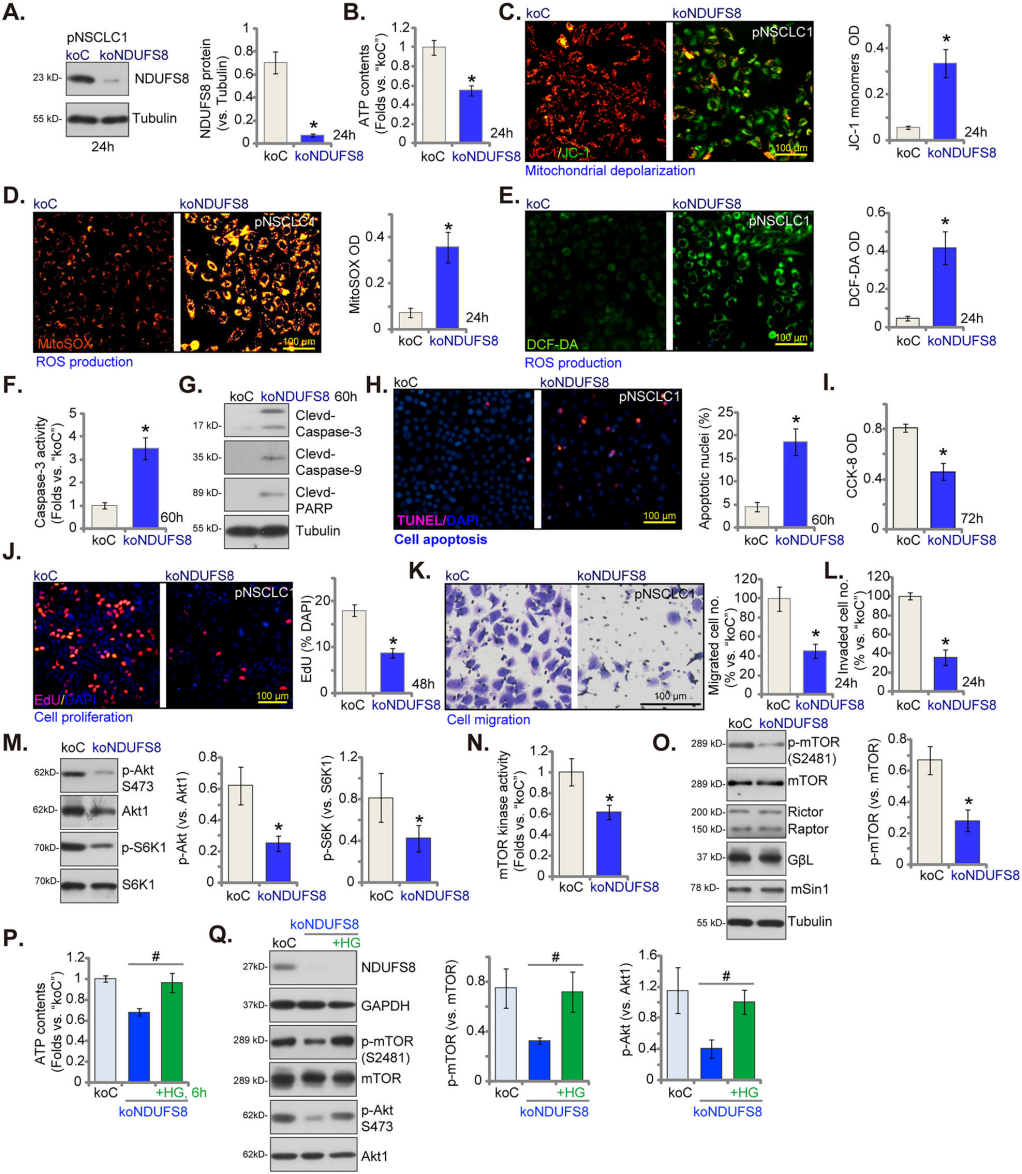
NDUFS8 knockout exerts significant anti-NSCLC cell activity. The primary human NSCLC cells, pNSCLC1, were engineered to stably express lentivirus-packed Cas9 construct plus the CRISPR/Cas9-NDUFS8-KO construct (“koNDUFS8”), while control cells express lentivirus-packed Cas9 construct plus CRISPR/Cas9-control construct (“koC”). Following cultivation of indicated time periods, NDUFS8 protein expression was measured (**A**). ATP levels were quantified (**B**), with mitochondrial depolarization evaluated based on JC-1 monomers’ intensity (**C**); ROS production was measured by MitoSOX and DCF-DA dyes (**D**, **E**). The Caspase-3 activity (**F**), expression of apoptosis-associated proteins (**G**) and cell apoptosis (via quantifying nuclear TUNEL ratio, **H**) were tested. Cell viability and proliferation were examined via CCK-8 (**I**) and nuclear EdU staining (**J**) assays respectively, with cell migration (**K**) and invasion (**L**) measured via “Transwell” chambers; Expression of listed proteins was shown (**M**, **O**), with the mTOR kinase activity measured (**N**). The koNDUFS8 pNSCLC-1 cells were maintained in regular medium (5.5 mM glucose) or switched to high-glucose (25 mM, “HG”) medium. koC control cells remained in the regular medium. After 6 h of cultivation, cellular ATP levels were measured (**P**), and expression of the listed proteins was analyzed (**Q**). Error bars in the figures represent the mean ± standard deviation (SD), with statistical significance (*P < 0.05) when comparing “koC” cells. ^**#**^ *P* < 0.05 (***P***, **Q**).The experiments depicted in this figure were independently repeated five times (*n* = 5) and consistently yielded similar results. Scale bar = 100 μm.

To further support the role of NDUFS8 in NSCLC cells, CRISPR/Cas9 strategy was employed to knockout (KO) NDUFS8. Specifically, a lentiviral CRISPR/Cas9-NDUFS8-KO construct encoding sgRNA sequence against *NDUFS8* was transduced to Cas9-expressing pNSCLC1 cells. After puromycin-based selection and *NDUFS8* KO verification, the single stable NDUFS8 KO cells were established, namely koNDUFS8 cells. As compared to pNSCLC1 cells with the lentiviral CRISPR/Cas9-KO control construct with non-sense sgRNA (“koC”), NDUFS8 protein expression was substantially decreased in koNDUFS8 cells (Fig. 7A), and ATP contents were reduced (Fig. 7B). NDUFS8 KO also disrupted mitochondrial functions and caused mitochondrial depolarization. The latter was again evidenced by accumulation of JC-1 green monomers (Fig. 7C). Increased ROS production was also detected in koNDUFS8 cells, supported by the increased MitoSOX red intensity (Fig. 7D) and DCF-DA green intensity (Fig. 7E). Significant apoptosis activation was detected in koNDUFS8 pNSCLC1 cells. The Caspase-3 activity (Fig. 7F) and cleavages of Caspase-3, Caspase-9 and PARP1 (Fig. 7G) were enhanced in koNDUFS8 cells. Moreover, TUNEL-positively stained nuclei ratio was increased in NDUFS8 KO pNSCLC1 cells (Fig. 7H). Cell viability, or CCK-8 OD was decreased (Fig. 7I). The proliferation of pNSCLC1 cells, tested by EdU incorporation, was also inhibited after NDUFS8 KO (Fig. 7J). Additionally, the in vitro cell migration (Fig. 7K) and invasion (Fig. 7L) were largely inhibited in koNDUFS8 pNSCLC1 cells. NDUFS8 KO also suppressed Akt-mTOR signaling in pNSCLC-1 cells, as evidenced by significantly decreased levels of p-Akt and p-S6K1 (Fig. 7M). The mTOR kinase activity (Fig. 7N) as well as the mTOR phosphorylation at Ser-2481 (Fig. 7O) were also decreased in NDUFS8 KO pNSCLC1 cells. Expression levels of mTOR complex components, including mTOR, Rictor, Raptor, GβL and mSin1 were indifferent after NDUFS8 KO (Fig. 7O). In koNDUFS8 pNSCLC1 cells, the transition to high glucose (25 mM, “HG”) medium resulted in the restoration of ATP levels (Fig. 7P) without affecting NDUFS8 expression (Fig. 7Q). Importantly, this change also reinstated the phosphorylations of mTOR (Ser-2481) and Akt in pNSCLC1 cells (Fig. 7Q). These results further support the notion that the decreased ATP levels resulting from NDUFS8 depletion possibly lead to reduced mTOR kinase activity, subsequently inhibiting the phosphorylation of Akt and S6K in NSCLC cells.

We next propose that mitochondrial inhibitors should also produce substantial anti-NSCLC cell activity. Rotenone functions by inhibiting the mitochondrial complex I in the electron transport chain, leading to decreased ATP production [53]. Antimycin A is an antibiotic produced by the bacterium *Streptomyces griseus* and inhibits mitochondrial respiration by blocking the electron transport chain at complex III [54]. In pNSCLC1 primary cancer cells, treatment with either rotenone or antimycin A led to mitochondrial depolarization (Fig. S3A), ATP depletion (Fig. S3B), and ROS production (MitoSOX intensity increasing, Fig. S3C), resulting in impaired cell proliferation (Fig. S3D), diminished viability (Fig. S3E), and reduced migration (Fig. S3F). Additionally, these two inhibitors activated apoptotic pathways, evidenced by an increased ratio of TUNEL-positive nuclei (Fig. S3G) and induced cell death, as demonstrated by enhanced Trypan blue staining (Fig. S3H).

### NDUFS8 overexpression exerts pro-cancerous activity in NSCLC cells

**Fig. 8:**
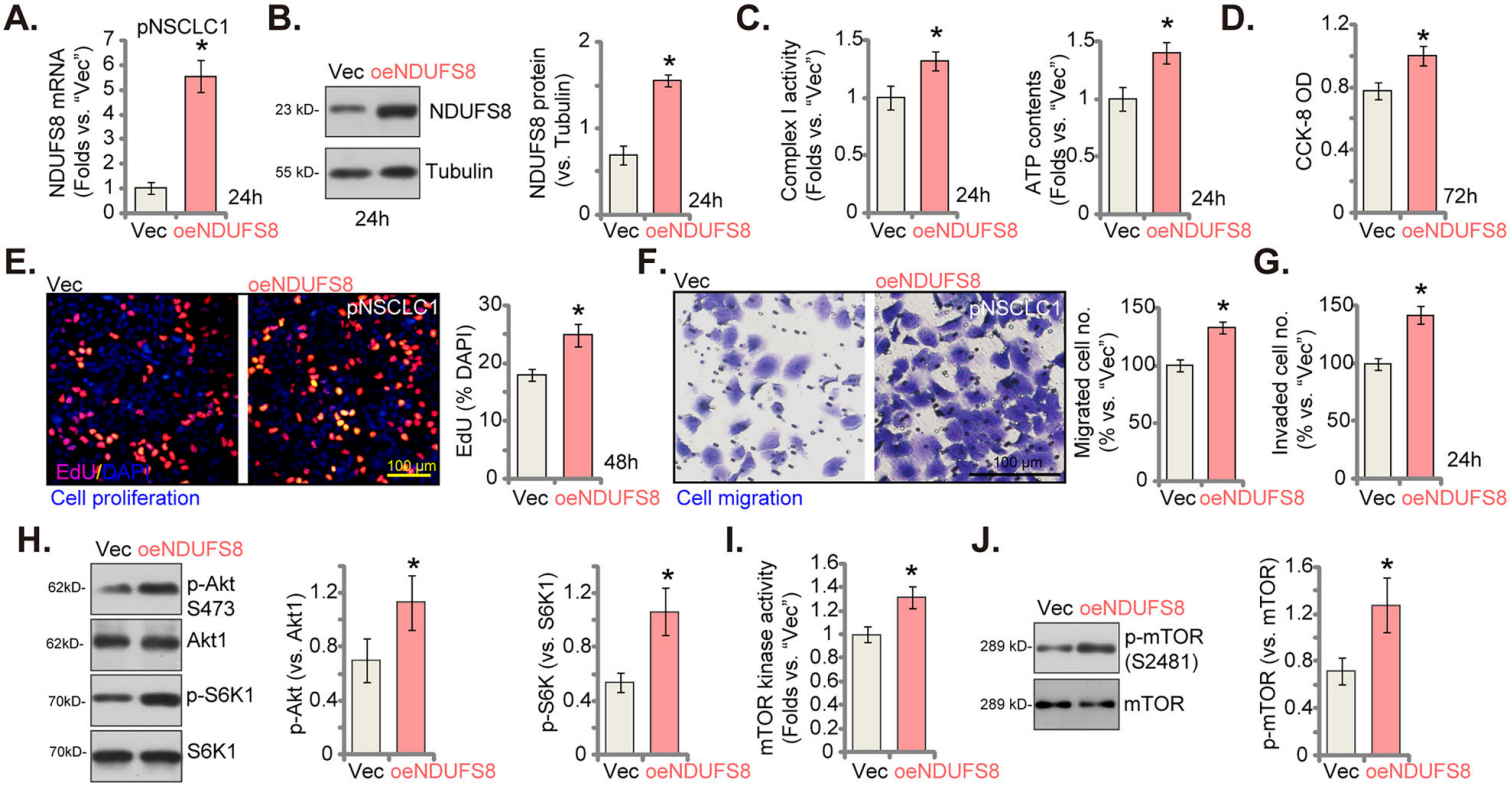
NDUFS8 overexpression exerts pro-cancerous activity in NSCLC cells. The primary human NSCLC cells, pNSCLC1, were engineered to stably express lentivirus-packed NDUFS8-overexpressing construct (“oeNDUFS8”), while control cells express lentivirus-packed empty vector (“Vec”). Following cultivation of indicated time periods, *NDUFS8* mRNA (**A**) and protein (**B**) expression was measured. The mitochondrial complex I activity and cellular ATP contents (**C**) were tested. Cell viability was assessed via CCK-8 assay (**D**). Cell proliferation was measured via nuclear EdU staining (**E**), with cell migration (**F**) and invasion (**G**) measured via “Transwell” chambers; Expression of listed proteins was also shown (**H**, **J**). The mTOR kinase activity was also measured (**I**). Error bars in the figures represent the mean ± standard deviation (SD), with statistical significance (*P < 0.05) when comparing “Vec” cells. The experiments depicted in this figure were independently repeated five times (*n* = 5) and consistently yielded similar results. Scale bar = 100 μm.

We next hypothesized that ectopic NDUFS8 overexpression shall exert pro-cancerous activity and promote NSCLC cell progression. Thus, a lentivirus-packaged NDUFS8 overexpression construct was stably transduced to pNSCLC1 cells, and stable cells, oeNDUFS8, were formed after puromycin-based selection. As compared to the control cells with the empty vector (“Vec”), *NDUFS8* mRNA (Fig. 8A) and protein (Fig. 8B) expression was significantly increased in oeNDUFS8 pNSCLC1 cells. With NDUFS8 overexpression, the mitochondrial complex I activity and the cellular ATP contents were both significantly increased (Fig. 8C). NDUFS8 overexpression also augmented pNSCLC1 cell proliferation, increasing CCK-8 OD (Fig. 8D) and EdU-positive nuclei ratio (Fig. 8E). The pNSCLC1 cell in vitro migration (Fig. 8F) and invasion (Fig. 8G), tested via “Transwell” and “Matrigel Transwell” assays respectively, were also accelerated after ectopic NDUFS8 overexpression. In oeNDUFS8 pNSCLC1 cells, a significant increase in p-Akt and p-S6K1 levels was observed, indicative of Akt-mTOR pathway hyperactivation (Fig. 8H). The mTOR kinase activity (Fig. 8I) and the mTOR phosphorylation at Ser-2481 (Fig. 8J) were both significantly increased in oeNDUFS8 pNSCLC1 cells. These results supported that NDUFS8 overexpression exerted pro-cancerous activity in NSCLC cells, enhancing mitochondrial function and facilitating cell proliferation and motility.

### NDUFS8 depletion sensitizes ionizing radiation-induced cytotoxicity in primary NSCLC cells

**Fig. 9:**
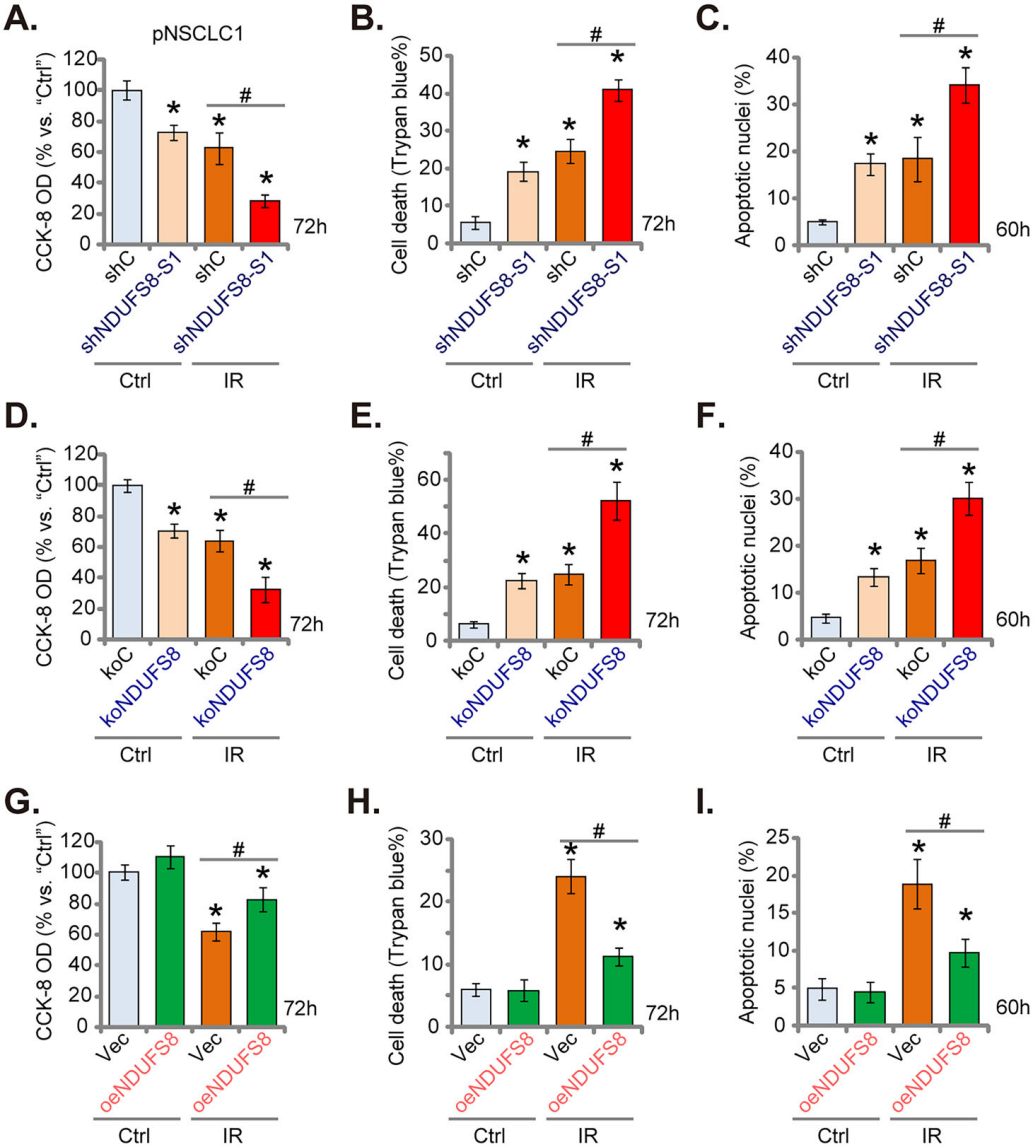
NDUFS8 depletion sensitizes ionizing radiation-induced cytotoxicity in primary NSCLC cells. The primary human NSCLC cells, pNSCLC1, stably expressing lentivirus-packed shNDUFS8-S1, the scramble control shRNA (“shC”) (**A**–**C**), lentivirus-packed Cas9 construct plus the CRISPR/Cas9-NDUFS8-KO construct (“koNDUFS8”) or the control construct (“koC”) (**D**–**F**), lentivirus-packed NDUFS8-overexpressing construct (“oeNDUFS8”) or the empty vector (“Vec”) (**G**–**I**) were treated with or without ionizing radiation (IR, 4 Gy) for indicated time periods, cell viability, death and apoptosis were tested via CCK8 (**A**, **D**, **G**), Trypan blue staining (**B**, **E**, **H**) and TUNEL nuclear staining (**C**, **F**, **I**) assays, respectively, with results quantified. “Ctrl” stands for the untreated control. Error bars in the figures represent the mean ± standard deviation (SD), with statistical significance (*P < 0.05) when comparing “Ctrl” cells. ^#^*P* < 0.05. The experiments depicted in this figure were independently repeated five times (*n* = 5) and consistently yielded similar results.

Understanding and addressing radiotherapy resistance mechanisms represent pivotal endeavors in the pursuit of improved outcomes for NSCLC patients [55, 56]. Here we found that ionizing radiation (IR) induced significant cytotoxicity in pNSCLC-1 cells, causing viability reduction (CCK8 OD) (Fig. 9A), cell death (tested via increased Trypan blue staining, Fig. 9B) and apoptosis (TUNEL-positive nuclei increasing, Fig. 9C). Importantly, shNDUFS8-S1-induced silencing of NDUFS8 exerted radio-sensitizing activity and intensified IR-induced cytotoxicity in pNSCLC-1 cells (Fig. 9A–C). Single shNDUFS8-S1 treatment also induced moderate but significant viability reduction (Fig. 9A), death (Fig. 9B) and apoptosis (Fig. 9C) in the primary cancer cells. In line with these findings, we found that IR-induced pNSCLC-1 cell death (Fig. 9D, E) and apoptosis (Fig. 9F) were also augmented in koNDUFS8 cells. Again, koNDUFS8 single treatment induced moderate cytotoxicity in pNSCLC-1 cells (Fig. 9D–F). Contrarily, in NDUFS8-overexpressing cells, oeNDUFS8, IR-caused viability reduction (Fig. 9G), cell death (Fig. 9H) and apoptosis (Fig. 9I) were largely ameliorated. These results supported that NDUFS8 could be an important radiotherapy resistance factor.

### NDUFS8 silencing inhibits NSCLC xenograft growth in nude mice

**Fig. 10:**
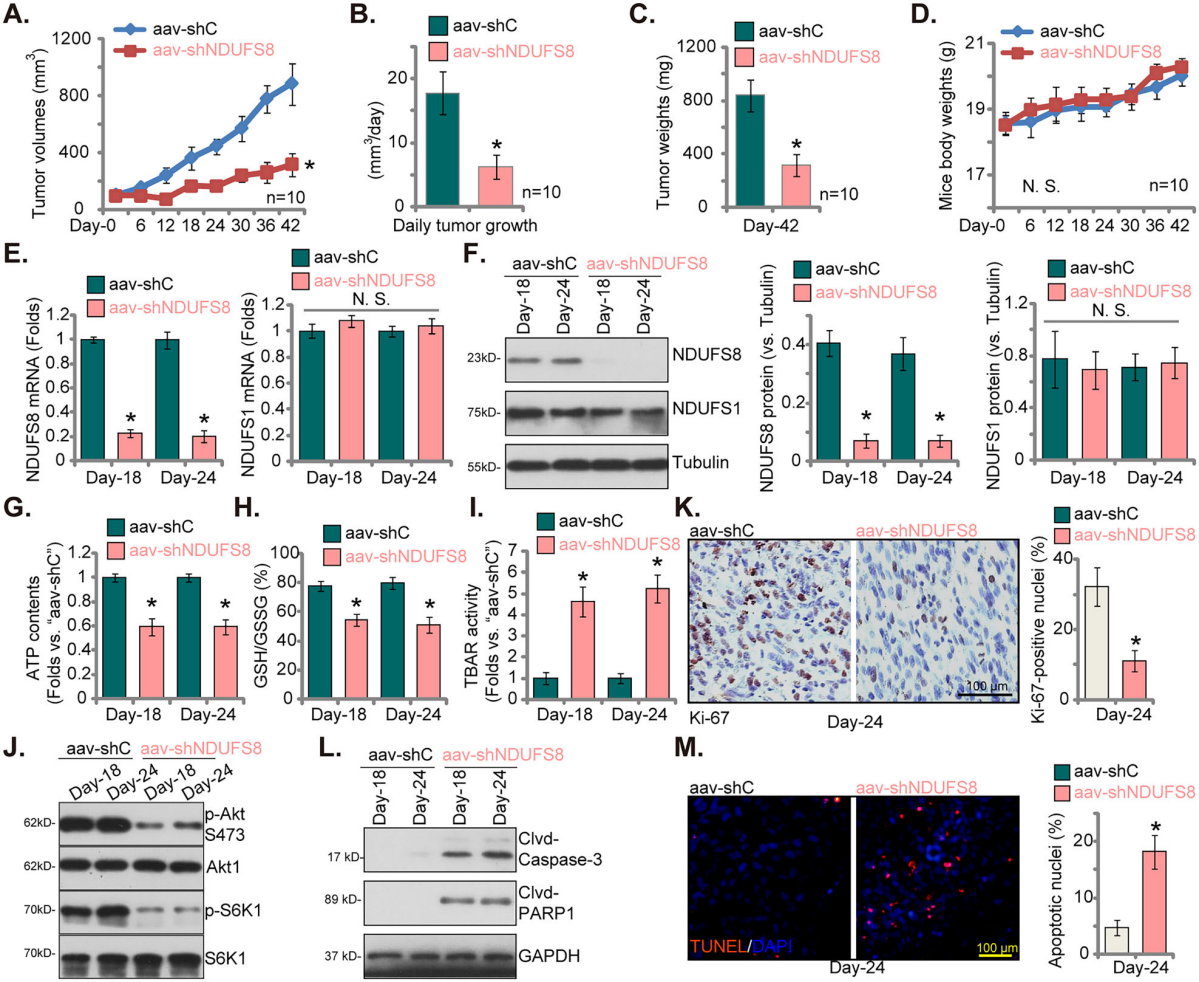
NDUFS8 silencing inhibits NSCLC xenograft growth in nude mice. The nude mice harboring pNSCLC-1 xenografts were subjected to intratumoral administration of adeno-associated virus (AAV) expressing either NDUFS8-specific shRNA (“aav-shNDUFS8”) or the control shRNA sequence (“aav-shC”), involving two rounds of virus injections, with a 48-hour interval between each injection. Tumor volumes (**A**) and the body weights of the mice (**D**) were monitored over a 42-day experimental period (“Day-0” to “Day-42”). The daily tumor growth rate was calculated (**B**). At the culmination of the 42-day experiment (Day-42), all pNSCLC-1 xenografts from both treatment groups were excised and their weights were recorded (**C**). On experimental Day-18 and Day-24, one pNSCLC-1 xenograft from each treatment group was carefully isolated to assess the expression levels of listed mRNAs and proteins (**E**, **F**, **J**, **L**). Additionally, ATP contents (**G**), GSG/GSSH ratio (**H**) and TBAR activity (**I**) were quantified. Immunohistochemistry staining (IHC) was performed to measure nuclear Ki-67 (**K**) within the pNSCLC-1 xenograft tissue sections. The xenograft sections were also subjected to fluorescence detection of TUNEL-positive nuclei (**M**). Error bars in the figures represent the mean ± standard deviation (SD), with statistical significance (*P < 0.05) when comparing “aav-shC” group. “N.S.” stands for non-statistically significant disparities (*P* > 0.05). In (**A**–**D**), ten mice were in each group (*n* = 10). For (**E**–**M**), five random tissue pieces in each xenograft were tested (*n* = 5). Scale bar = 100 μm.

To assess the potential influence of NDUFS8 on the in vivo growth of NSCLC cells, we conducted a xenograft study employing pNSCLC1 primary cancer cells. These cells were subcutaneously (*s.c*.) implanted into the flanks of nude mice at five million cells per mouse. The emergence of pNSCLC1 xenograft tumors was observed after a three-week period, designated as “Day-0.” Subsequently, intratumoral injections of adeno-associated virus (AAV) carrying NDUFS8 shRNA (“aav-shNDUFS8”) were administered to one group of nude mice, while a control group received injections of AAV containing scramble control shRNA (“aav-shC”). This virus administration was repeated at 48-hour intervals for two cycles. The results of the tumor growth curve demonstrated that aav-shNDUFS8 injection significantly hindered the growth of pNSCLC1 xenografts in nude mice (Fig. 10A), with the volumes of aav-shNDUFS8-treated xenografts markedly lower than those of aav-shC-treated xenografts (Fig. 10A). We calculated the estimated daily tumor growth rate (in mm^3^ per day, using the described formula [21, 34, 45]), reaffirming that aav-shNDUFS8 treatment effectively curtailed the growth of pNSCLC1 xenografts (Fig. 10B). On Day-42, all pNSCLC1 xenografts were harvested and individually weighed, revealing that those with aav-shNDUFS8 injection were substantially lighter compared to the aav-shC-treated xenografts (Fig. 10C). Importantly, no significant differences in the body weights of the mice were observed between the aav-shNDUFS8 and the aav-shC groups (Fig. 10D).

To achieve deeper insights into the signaling alterations associated with pNSCLC1 xenografts, we conducted an examination of tissue samples on experimental Days 18 and 24. One xenograft from each group (aav-shNDUFS8 and aav-shC) was carefully isolated for further analysis. Fresh tumor xenografts were processed, revealing a substantial reduction in *NDUFS8* mRNA and protein expression in pNSCLC1 xenograft tissues treated with aav-shNDUFS8 (Fig. 10E, F). Expression of the control gene, NDUFS1, was however unchanged (Fig. 10E, F). Additionally, we noted decreased ATP levels in pNSCLC1 xenograft tissues with aav-shNDUFS8 injection (Fig. 10G). A reduced GSH/GSSG ratio (Fig. 10H) and an elevated TBAR activity (Fig. 10I) provided compelling evidence of oxidative injury and lipid peroxidation in NDUFS8-silenced pNSCLC1 xenograft tissues. Akt-mTOR activation was inhibited in NDUFS8-silenced pNSCLC1 xenograft tissues, as the p-Akt and p-S6K1 levels were substantially reduced (Fig. 10J). Ki-67 staining in xenograft sections showed that aav-shNDUFS8 treatment decreased the Ki-67-positive nuclei ratio in pNSCLC1 xenografts, confirming its anti-proliferative activity (Fig. 10K). Moreover, increased cleavages of Caspase-3 and PARP1 were detected in pNSCLC1 xenograft tissues of aav-shNDUFS8 mice (Fig. 10L). The aav-shNDUFS8 treatment also induced apoptosis in pNSCLC1 xenograft tissues, as the TUNEL-positive nuclei ratio was significantly increased (Fig. 10M). Thus, the administration of aav-shNDUFS8 induced ATP depletion, oxidative damage, proliferation inhibition and apoptosis within pNSCLC1 xenograft tissues.

## Discussion

Despite advancements in NSCLC targeted therapies, issues remain including resistance, mutation suitability gaps, high costs, off-target effects, and acquired resistance, necessitating ongoing research to enhance efficacy and accessibility, and more importantly exploring novel molecular targets [9, 11–13, 57]. Very few studies have explored the expression and potential functions of NDUFS8 in human cancers. Su and colleagues have revealed high NDUFS8 expression is associated with poor overall survival in NSCLC patients [58]. When combined with NDUFS1, NDUFS8 becomes part of a powerful panel that serves as a novel prognostic predictor for NSCLC [58]. Our study demonstrated the essential role of NDUFS8 in preserving mitochondrial functions in NSCLC cells. Silencing or knocking out NDUFS8 in both primary and immortalized NSCLC cells disrupted mitochondrial functions, leading to decreased mitochondrial complex I activity, ATP depletion, mitochondrial depolarization, increased ROS production, and enhanced lipid peroxidation. Conversely, the ectopic overexpression of NDUFS8 resulted in elevated mitochondrial complex I activity and ATP levels in primary NSCLC cells. ATP depletion, ROS production, and enhanced lipid peroxidation were also detected in pNSCLC1 xenografts with aav-shNDUFS8 treatment. Thus, preserving mitochondrial hyperfunctions may represent a crucial mechanism underlying the progression of NSCLC cells driven by NDUFS8.

The findings of this study suggest that NDUFS8 represents a promising therapeutic target for NSCLC. TCGA database reveals that NDUFS8 is consistently overexpressed in NSCLC and is strongly correlated with poor overall survival, elevated N stages, and advanced pathological stages. Elevated NDUFS8 expression was consistently observed in various local human NSCLC tissues and multiple NSCLC cells. scRNA-seq results reveal the upregulation of NDUFS8 in cancerous cells of NSCLC. Silencing or knocking out NDUFS8 induced apoptosis and significantly suppressed cell viability, proliferation, and motility across primary and immortalized NSCLC cells. Conversely, the ectopic overexpression of NDUFS8 using a lentivirus-based vector augmented NSCLC cell proliferation and motility. The in vivo studies showed that intratumoral administration of aav-shNDUFS8 markedly attenuated the growth of subcutaneous primary NSCLC xenografts in nude mice. These results underscore the potential therapeutic value of targeting NDUFS8 in NSCLC treatment.

Akt-mTOR overactivation is vital for the development and progression of NSCLC [59–61]. Our findings demonstrate that NDUFS8 is important in activating the Akt-mTOR signaling pathway in NSCLC cells. In vitro studies demonstrated that the knockdown or knockout of NDUFS8 in pNSCLC1 cells markedly reduced the phosphorylation of Akt and S6K1. Conversely, the overexpression of NDUFS8 resulted in increased phosphorylation of these proteins, thereby providing compelling evidence for the role of NDUFS8 in the activation of the Akt-mTOR pathway. This observation was further validated in vivo, as NDUFS8 silencing in xenograft models resulted in a substantial decrease in phosphorylated Akt and S6K1 levels. Importantly, re-establishing Akt activation by caAkt1 not only ameliorated the reduction in cell viability, proliferation, and migratory capacity but also attenuated apoptosis and cell death associated with NDUFS8 silencing. These results strongly suggest that NDUFS8 is a key regulator of the Akt-mTOR pathway in NSCLC. Mechanistically, our findings indicate that NDUFS8 depletion lowers ATP levels in NSCLC cells, which attenuates mTOR kinase activity, thereby suppressing phosphorylation of Akt and S6K.

Radiotherapy constitutes a frequently employed therapeutic strategy for the management of NSCLC, involving the precise and controlled irradiation of malignant pulmonary tissue [55, 56]. Its application extends across primary treatment and combinatory approaches in conjunction with surgery, chemotherapy, and immunotherapy, contingent on the disease’s staging and specific attributes [55, 56]. Radio-resistance in NSCLC involves a complex interplay of mechanisms, including enhanced DNA damage repair, protection from the tumor microenvironment, the presence of radio-resistant cancer stem cells, hypoxia, dysregulated signaling pathways, epithelial-mesenchymal transition (EMT), and immune evasion [55, 56]. Ongoing investigations are centered on multifaceted approaches, incorporating targeted therapeuties and immunotherapies, in a concerted effort to bolster the efficacy of radiotherapy and improve clinical outcomes in NSCLC patients [55, 56]. The current investigation demonstrates the potential significance of NDUFS8 as a pivotal contributor to radio-resistance in NSCLC. Silencing or KO of NDUFS8 in patient-derived NSCLC cells heightened the cytotoxicity induced by IR, while conversely, overexpression of NDUFS8 ameliorated this IR-induced cytotoxicity. Further studies will be needed to explore the underlying mechanism of NDUFS8 overexpression in radio-resistance within NSCLC cells.

## Supplementary information

Original data set

Figure S1-S3

## Data Availability

All necessary data are provided in the paper and Supplementary Materials.
