# Supplementary material for: NADH:ubiquinone oxidoreductase core subunit S8 expression and functional significance in non-small cell lung cancer
Source: Cell Death Dis. 2025 Apr 21;16(1):321. doi: 10.1038/s41419-025-07638-5 (PMC12012183; doi:10.1038/s41419-025-07638-5)

Figure S1. The uncropped blotting images of the study.

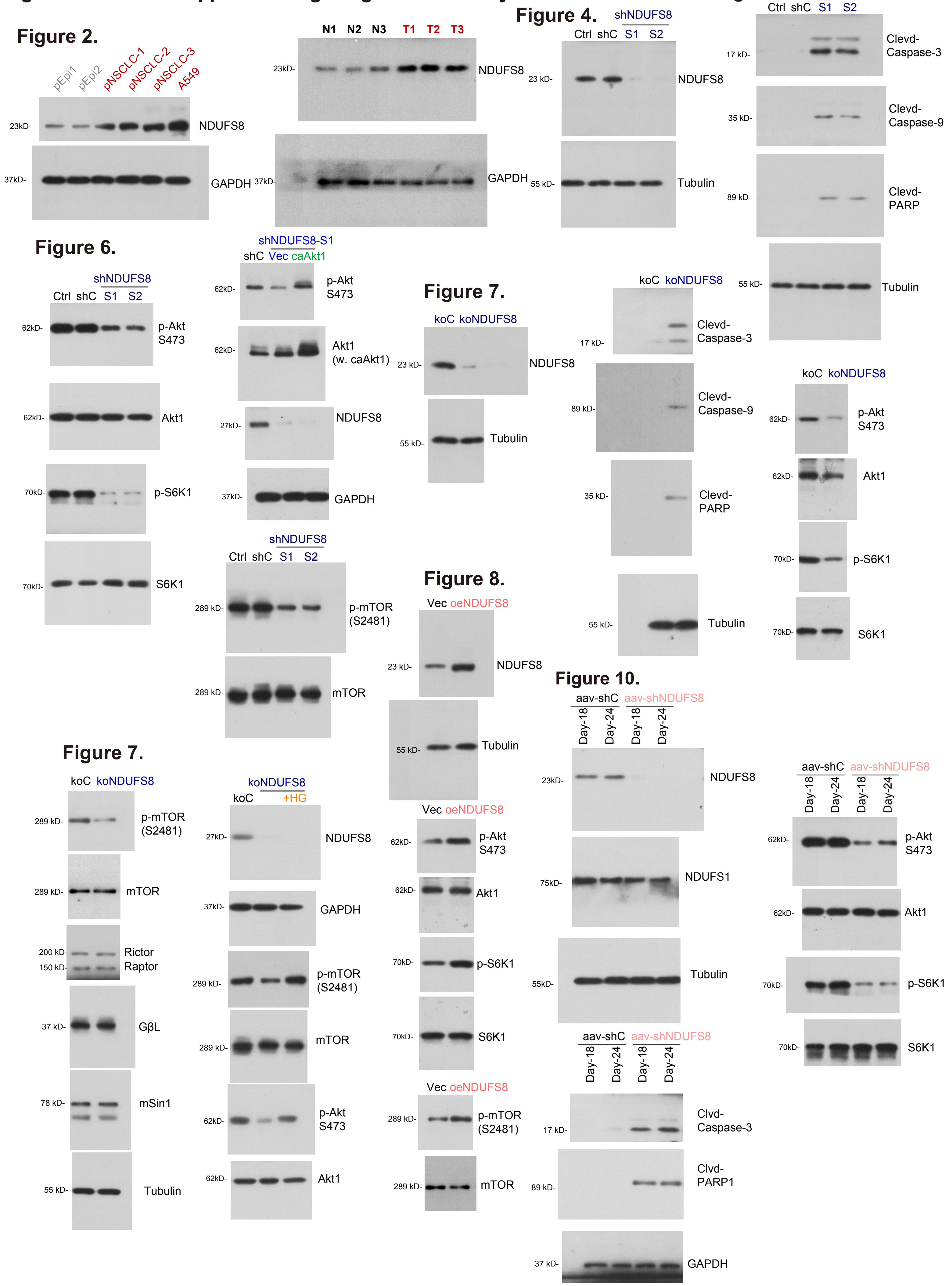

Supplement: Supplementary file 1 — Original data set [file 41419_2025_7638_MOESM1_ESM.pdf]
