# Supplementary material for: NADH:ubiquinone oxidoreductase core subunit S8 expression and functional significance in non-small cell lung cancer
Source: Cell Death Dis. 2025 Apr 21;16(1):321. doi: 10.1038/s41419-025-07638-5 (PMC12012183; doi:10.1038/s41419-025-07638-5)
Supplement: Supplementary file 2 — Figure S1-S3 [file 41419_2025_7638_MOESM2_ESM.pdf]

Figure S1. The uncropped blotting images of the study.

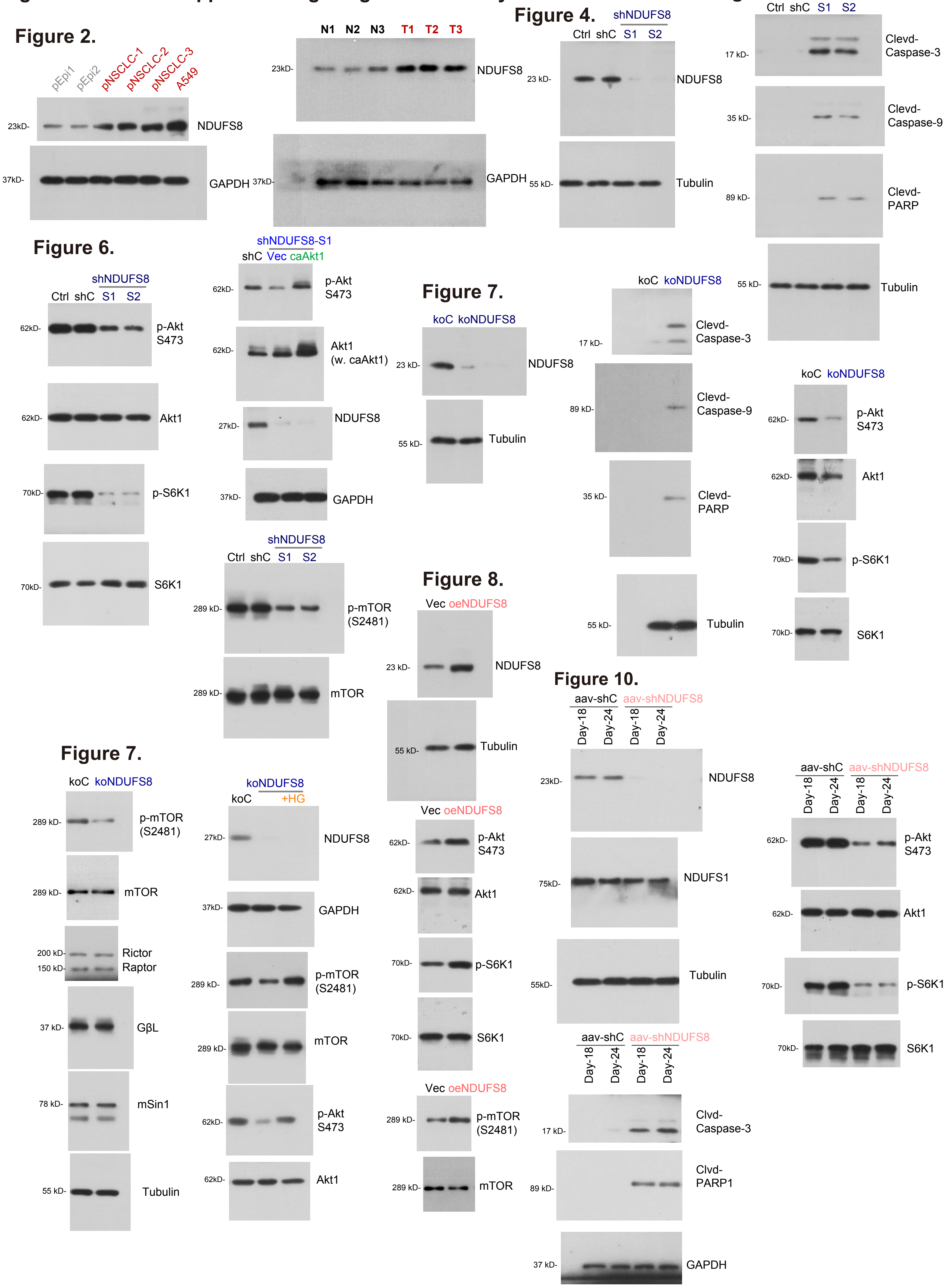

Figure S2

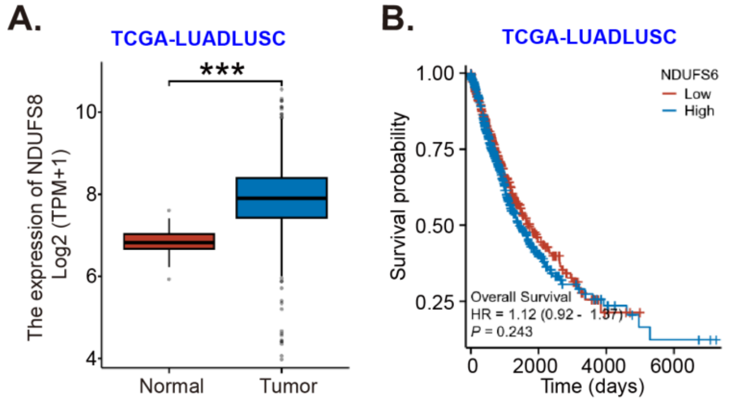

Figure S2. The Cancer Genome Atlas (TCGA) lung adenocarcinoma (LUAD) lung squamous cell carcinoma (LUSC) cohort (combining both LUAD and LUSC) shows *NDUFS8* expression in NSCLC tissues (“Tumor”) and normal lung tissues (“Normal”) (**A**). The Kaplan-Meier survival analyses show that the association between *NDUFS8* expression and the overall survival of NSCLC patients (**B**). Transcripts Per Million (TPM). *** $P < 0.001$.

Figure S3

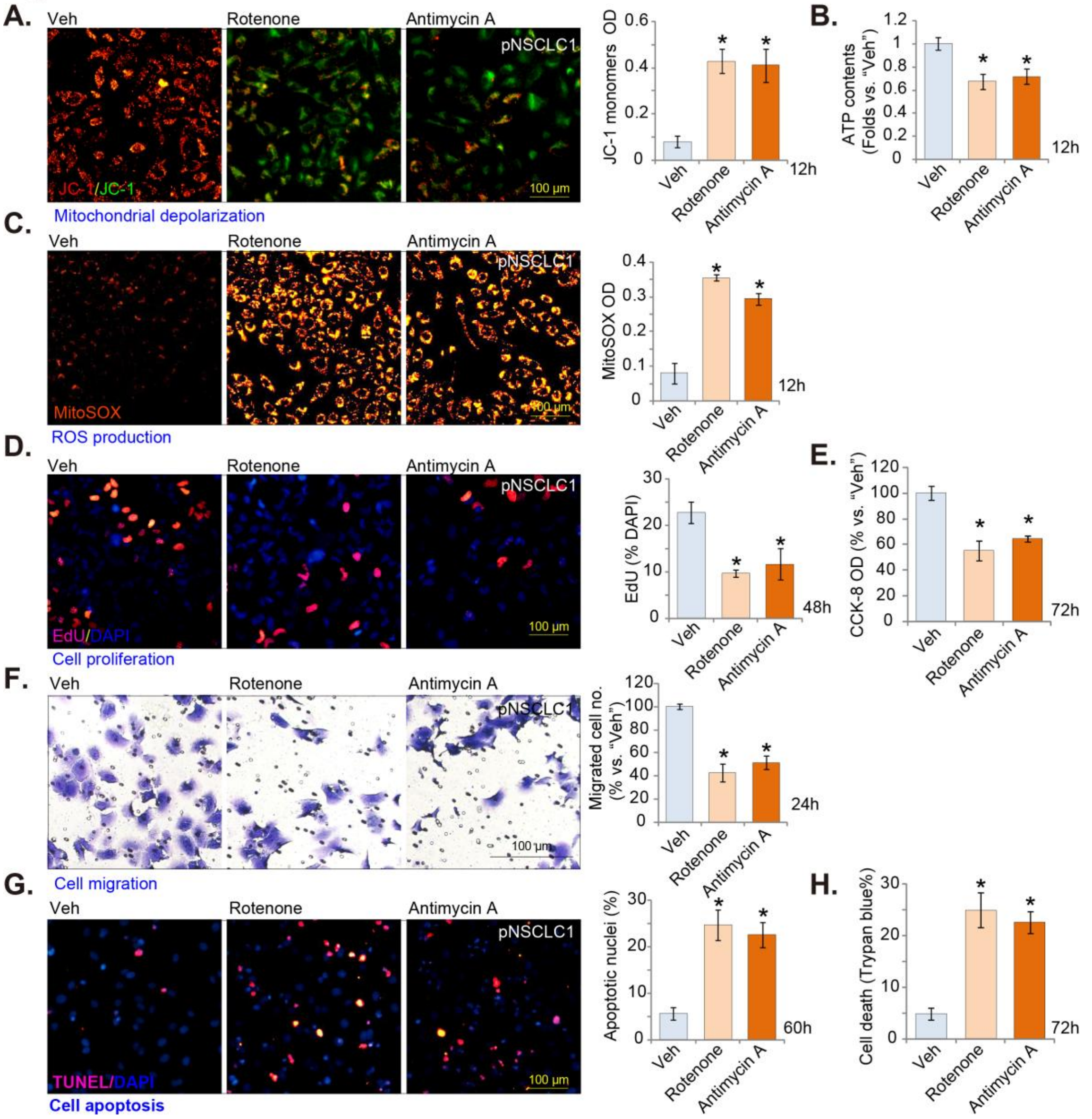

Figure S3. The primary human NSCLC cells, pNSCLC1, were treated with rotenone (10 μ M), Antimycin A (5 M) or vehicle control (0.1 % DMSO, "Veh") for designated time periods, cellular functions, including mitochondrial depolarization (evaluated based on JC-1 monomers' intensity, **A**), cellular ATP contents (**B**), ROS production (tested via MitoSOX intensity, **C**) were measured; Cell proliferation, viability and migration were examined via nuclear EdU staining (**D**), CCK-8 OD (**E**), and "Transwell" (**F**) assays, respectively. Cell apoptosis (via quantifying nuclear TUNEL ratio, **G**) and cell death (Trypan blue staining assays, **H**) were also tested. Error bars in the figures represent the mean ± standard deviation (SD), with statistical significance (* $P < 0.05$) when comparing "Veh" treatment. The experiments depicted in this figure were independently repeated five times (n=5) and consistently yielded similar results. Scale bar = 100 μm.
